# Long-term outcomes of young, node-negative, chemotherapy-naïve, triple-negative breast cancer patients according to *BRCA1* status

**DOI:** 10.1186/s12916-023-03233-7

**Published:** 2024-01-09

**Authors:** Yuwei Wang, Gwen M. H. E. Dackus, Efraim H. Rosenberg, Sten Cornelissen, Leonora W. de Boo, Annegien Broeks, Wim Brugman, Terry W. S. Chan, Paul J. van Diest, Michael Hauptmann, Natalie D. ter Hoeve, Olga I. Isaeva, Vincent M. T. de Jong, Katarzyna Jóźwiak, Roelof J. C. Kluin, Marleen Kok, Esther Koop, Petra M. Nederlof, Mark Opdam, Philip C. Schouten, Sabine Siesling, Charlaine van Steenis, Adri C. Voogd, Willem Vreuls, Roberto F. Salgado, Sabine C. Linn, Marjanka K. Schmidt

**Affiliations:** 1 Division of Molecular Pathology, Netherlands Cancer Institute, Plesmanlaan 121, 1066 CX Amsterdam, The Netherlands; 2 Department of Pathology, University Medical Center Utrecht, Utrecht, The Netherlands; 3 Division of Pathology, Netherlands Cancer Institute, Amsterdam, The Netherlands; 4 Core Facility Molecular Pathology and Biobanking, Netherlands Cancer Institute, Amsterdam, The Netherlands; 5 Genomics Core Facility, Netherlands Cancer Institute, Amsterdam, The Netherlands; 6 Institute of Biostatistics and Registry Research, Brandenburg Medical School Theodor Fontane, Neuruppin, Germany; 7 Division of Tumor Biology and Immunology, Netherlands Cancer Institute, Amsterdam, The Netherlands; 8 Division of Molecular Oncology and Immunology, Netherlands Cancer Institute, Amsterdam, The Netherlands; 9 Department of Medical Oncology, Netherlands Cancer Institute, Amsterdam, the Netherlands; 10 Department of Pathology, Gelre Ziekenhuizen, Apeldoorn, The Netherlands; 11 Department of Research and Development, Netherlands Comprehensive Cancer Organization, Utrecht, The Netherlands; 12 Department of Health Technology and Services Research, Technical Medical Centre, University of Twente, Enschede, The Netherlands; 13 Department of Epidemiology, Maastricht University, Maastricht, The Netherlands; 14 Department of Pathology, Canisius Wilhelmina Ziekenhuis, Nijmegen, The Netherlands; 15 Department of Pathology, GZA-ZNA Hospitals, Antwerp, Belgium; 16 Division of Research, Peter MacCallum Cancer Center, Melbourne, Australia; 17 Department of Clinical Genetics, Leiden University Medical Center, Leiden, The Netherlands

**Keywords:** *BRCA1* status, Tumor-infiltrating lymphocytes, Triple-negative breast cancer, Chemotherapy-naïve, Long-term outcomes, Risk classification

## Abstract

**Background:**

Due to the abundant usage of chemotherapy in young triple-negative breast cancer (TNBC) patients, the unbiased prognostic value of *BRCA1*-related biomarkers in this population remains unclear. In addition, whether *BRCA1*-related biomarkers modify the well-established prognostic value of stromal tumor-infiltrating lymphocytes (sTILs) is unknown. This study aimed to compare the outcomes of young, node-negative, chemotherapy-naïve TNBC patients according to *BRCA1* status, taking sTILs into account.

**Methods:**

We included 485 Dutch women diagnosed with node-negative TNBC under age 40 between 1989 and 2000. During this period, these women were considered low-risk and did not receive chemotherapy. *BRCA1* status, including pathogenic germline *BRCA1* mutation (g*BRCA1*m), somatic *BRCA1* mutation (s*BRCA1*m), and tumor *BRCA1* promoter methylation (*BRCA1*-PM), was assessed using DNA from formalin-fixed paraffin-embedded tissue. sTILs were assessed according to the international guideline. Patients’ outcomes were compared using Cox regression and competing risk models.

**Results:**

Among the 399 patients with *BRCA1* status, 26.3% had a g*BRCA1*m, 5.3% had a s*BRCA1*m, 36.6% had tumor *BRCA1-*PM, and 31.8% had *BRCA1*-non-altered tumors. Compared to *BRCA1*-non-alteration, g*BRCA1*m was associated with worse overall survival (OS) from the fourth year after diagnosis (adjusted HR, 2.11; 95% CI, 1.18–3.75), and this association attenuated after adjustment for second primary tumors. Every 10% sTIL increment was associated with 16% higher OS (adjusted HR, 0.84; 95% CI, 0.78–0.90) in g*BRCA1*m, s*BRCA1*m, or *BRCA1*-non-altered patients and 31% higher OS in tumor *BRCA1*-PM patients. Among the 66 patients with tumor *BRCA1*-PM and ≥ 50% sTILs, we observed excellent 15-year OS (97.0%; 95% CI, 92.9–100%). Conversely, among the 61 patients with g*BRCA1*m and < 50% sTILs, we observed poor 15-year OS (50.8%; 95% CI, 39.7–65.0%). Furthermore, g*BRCA1*m was associated with higher (adjusted subdistribution HR, 4.04; 95% CI, 2.29–7.13) and tumor *BRCA1*-PM with lower (adjusted subdistribution HR, 0.42; 95% CI, 0.19–0.95) incidence of second primary tumors, compared to *BRCA1*-non-alteration.

**Conclusions:**

Although both g*BRCA1*m and tumor *BRCA1*-PM alter *BRCA1* gene transcription, they are associated with different outcomes in young, node-negative, chemotherapy-naïve TNBC patients. By combining sTILs and *BRCA1* status for risk classification, we were able to identify potential subgroups in this population to intensify and optimize adjuvant treatment.

**Supplementary Information:**

The online version contains supplementary material available at 10.1186/s12916-023-03233-7.

## Background

Pathogenic germline *BRCA1* mutations (g*BRCA1*m) predispose women to breast cancer, especially triple-negative breast cancer (TNBC) [1]. Approximately 8.5 to 16% of unselected TNBC patients carry a pathogenic g*BRCA1*m [2–5]. This percentage is higher in those who are diagnosed at a younger age, ranging from 20 to 36% [2, 6–8]. Besides g*BRCA1*m, somatic *BRCA1* mutations (s*BRCA1*m) and *BRCA1* promoter methylation (*BRCA1*-PM) also alter the transcription of the *BRCA1* gene. Since altered *BRCA1* transcription hampers the homologous recombination pathway, leading to unrepaired DNA double-strand breaks and genomic instability, the affected tumors often present a typical profile of genomic aberrations [9–11]. In this study, we defined tumors with the typical genomic aberrations, which resemble the aberrations caused by g*BRCA1*m as *BRCA1*-like tumors [12]. In addition, increased genomic instability is suggested to promote anti-tumor immune response [13], which might be reflected by the abundance of tumor-infiltrating lymphocytes (TILs) in g*BRCA1*m or *BRCA1*-like tumors. Several studies have demonstrated that high TILs were associated with improved prognosis of TNBC patients [14–17]. However, whether TILs are more enriched in g*BRCA1*m or *BRCA1*-like tumors remains in dispute [18–21]. In addition, the prognostic value of TILs in g*BRCA1*m patients or in patients with other *BRCA1*-altered tumors is unclear.

Moreover, g*BRCA1*m or *BRCA1*-like tumors often present with aggressive phenotypes [22, 23] and are hypothesized to be associated with a worse prognosis compared to germline *BRCA1* wild-type (g*BRCA1*wt) or non-*BRCA1*-like tumors. However, many studies observed that in chemotherapy-treated TNBC patients, those with g*BRCA1*m or *BRCA1*-like tumors had equivalent or even better survival compared to those with g*BRCA1*wt or non-*BRCA1*-like tumors [5, 6, 24–27]. This suggests that chemotherapy might obscure the worse survival of patients with g*BRCA1*m or *BRCA1-*like tumors [28]. However, robust evidence from studies with large sample sizes and minimal indication bias is scarce.

Few studies have directly compared the outcomes of patients with different *BRCA1*-related biomarkers, let alone in women who did not receive (neo)adjuvant chemotherapy. Investigating the disease course of these tumors, independent of the curative effects of chemotherapy, will help to understand the true prognostic value of *BRCA1*-related biomarkers. This study aimed to compare long-term outcomes of young, node-negative, (neo)adjuvant chemotherapy-naïve TNBC patients according to g*BRCA1*m, s*BRCA1*m, or tumor *BRCA1-*PM, or according to *BRCA1*-like status, taking into account TILs and other established clinicopathological characteristics.

## Methods

### Study population

**Fig. 1:**
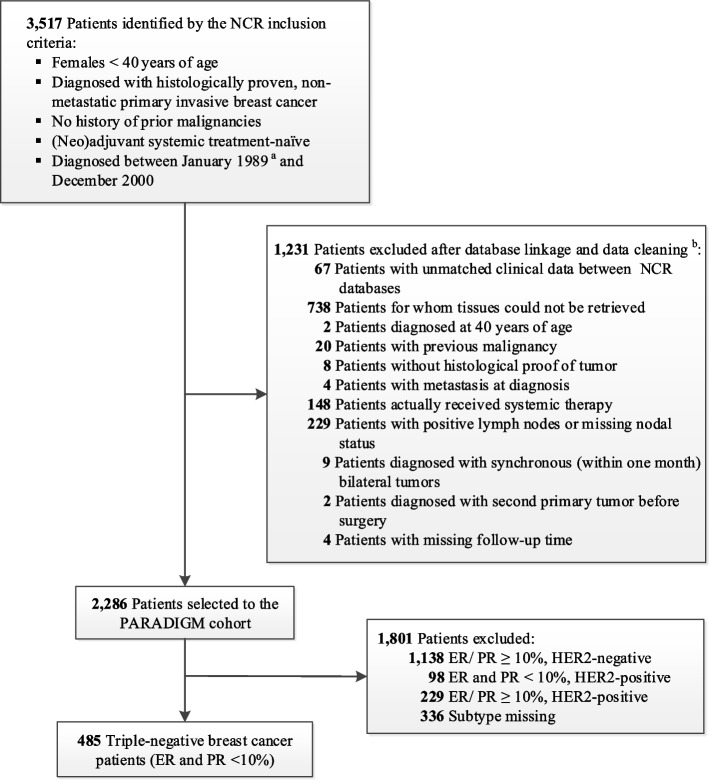
Selection of young, chemotherapy-naïve triple-negative breast cancer patients. *Abbreviations*: NCR, Netherlands Cancer Registry; ER, estrogen receptor; PR, progesterone receptor; HER2, human epidermal growth factor receptor 2. ^a^The NCR provides nationwide registry since 1989. ^b^The exclusion steps are in subsequent order

All women with TNBC (*n* = 485; age at diagnosis ranged from 22 to 39 years) were selected from the nationwide, population-based PARADIGM cohort. The study design has been described elsewhere [29]. Briefly, the PARADIGM cohort included all (neo)adjuvant systemic therapy-naïve patients diagnosed under age 40 between 1989 and 2000 with non-metastatic, invasive breast cancer from the Netherlands Cancer Registry (Fig. 1). The final selection of the PARADIGM cohort only included node-negative patients, since adjuvant treatment allocation before 2000 was mostly based on nodal status [30]. Stromal TILs (sTILs) were assessed according to the international guideline [31] by an experienced pathologist using hematoxylin and eosin-stained, formalin-fixed, paraffin-embedded whole slides, as previously described [16]. Information on distant recurrences and incidence of second primary tumors was collected until June 2014; information on death was collected until January 2018. Among the TNBC patients, eight were lost to the follow-up. Further details are provided in Additional file 1: Supplementary Methods [12, 32–38].

### Assessment of BRCA1-related biomarkers

*BRCA1* status was determined according to the g*BRCA1*m, s*BRCA1*m, and tumor *BRCA1*-PM status. Tumor DNA and normal DNA were isolated from archived formalin-fixed, paraffin-embedded tumor and normal tissues, respectively, at the NKI. Multiplicom (Niel, Belgium), now incorporated into Agilent (Carpinteria, CA, USA), analyzed single-nucleotide variants (SNVs) and small insertions or deletions (indels) using the NGS SureSelect and/or SureMASTR HRR kit (Agilent Technologies). The hg19 human reference genome was used for the alignment. The results were analyzed in Bench Lab NGS v4.3.5 (Agilent Technologies) by an expert clinical molecular geneticist. In this study, we only referred to (likely) pathogenic (class 4/class 5) variants as mutations [39]. Tumor *BRCA1*-PM was analyzed using methylation-specific multiplex ligation-dependent probe amplification at the NKI. Tumors with neither a *BRCA1* mutation (SNVs or indels) nor *BRCA1*-PM, or those with unknown *BRCA1* mutation and/or *BRCA1*-PM status were additionally analyzed for Dutch founder mutations, i.e., *BRCA1* exon 13 or 22 deletions, using deletion-specific PCR. Tumor *BRCA1* mutations were confirmed with tumor DNA and matched normal DNA, using Sanger sequencing at the NKI, and were classified as somatic or germline (Additional file 2: Fig. S1).

The *BRCA1*-like classifier [12] was used to classify tumors with or without *BRCA1*-like genomic aberrations, using copy number profiles, obtained with low-coverage whole-genome sequencing [34, 35]. All *BRCA1*-related biomarkers were assessed blinded to the clinical outcomes. See Additional file 1: Supplementary Methods for more information on all biomarkers assessed, including *BRCA1* mRNA expression levels.

### Statistical analysis

sTILs and other clinicopathological characteristics, *BRCA1*-like status, *BRCA1* mRNA expression, and treatment according to *BRCA1* status were compared using the Kruskal–Wallis tests (continuous outcomes) and chi-square or Fisher’s exact tests (categorical outcomes). Similarly, we compared the clinicopathological characteristics, *BRCA1* mRNA expression, and treatment according to *BRCA1*-like status using the aforementioned tests.

We assessed patients’ clinical outcomes, including overall survival (OS), distant recurrence-free survival (DRFS), and cumulative incidence of second primary tumors, stratified by *BRCA1* status and *BRCA1*-like status. The five germline *BRCA2* mutation carriers were excluded from clinical outcome analyses because previous studies have reported different associations of *BRCA2* versus *BRCA1* mutations with breast cancer prognosis [40–45]. Furthermore, the limited number of germline *BRCA2* mutation carriers precluded from providing valid estimates. For OS, follow-up started at diagnosis and ended at death due to any cause or was administratively censored at 15 years because events occurring after this period were unlikely to be related to the initial TNBC diagnosis. For DRFS, follow-up started at diagnosis and ended at distant recurrence or death, or was censored at the incidence of second primary tumors, the last day of event collection, or at 15 years, whichever came first. For cumulative incidence of second primary tumors, the follow-up started at diagnosis and ended at the second primary tumor, or was censored at distant recurrence or death, or the last day of event collection, or at 15 years, whichever came first.

Absolute OS and DRFS were derived using the Kaplan–Meier method. Survival rates for different *BRCA1* status were compared using log-rank tests. Hazard ratios (HR) for *BRCA1* status on OS and DRFS were calculated using univariable and multivariable Cox regression models with adjustment for sTILs, other clinicopathological characteristics, and treatment. The cumulative incidence of second primary tumors was calculated using a non-parametric approach [46], with distant recurrence and death as competing events. The incidences for different *BRCA1* status were compared using Gray’s tests. Subdistribution HRs for *BRCA1* status on second primary tumors were calculated using univariable and multivariable Fine and Gray competing risk models with adjustment for sTILs, other clinicopathological characteristics, and treatment. Distant recurrence and death were considered competing events. Cause-specific HRs were calculated using cause-specific Cox regression models in case the subdistribution HRs reflected an indirect association through the competing events [47].

The proportionality of hazards was examined using Schoenfeld residuals. In cases where the assumption was violated, an interaction term of the variable of interest and follow-up periods was added. To test if the association between sTILs and each clinical outcome differed across *BRCA1* status, we added interaction terms between *BRCA1* status and sTILs in multivariable models. Only the significant interaction terms were kept in the final model. To assess whether second primary tumors mediated the relationship between *BRCA1* status and OS, a time-varying covariate for second primary tumors was added in the multivariable model for OS.

Multiple imputation of missing values was performed using chained equations (MICE package, version 3.15.0, in R; see Additional file 1: Supplementary Methods). All regression models were performed using multiple-imputed data and cases with complete information separately. Sensitivity analyses were performed on patients with tumor ER and PR expression < 1% and on those diagnosed between 1989 and 1997, due to the recommendation of chemotherapy to some node-negative patients in the Netherlands after 1997. This led to a lower number of patients diagnosed after 1997 being included in this cohort. A post hoc sensitivity analysis was also conducted on patients with *BRCA1*-like tumors.

Detailed statistical analyses are described in Additional file 1: Supplementary Methods. All statistical tests were two-sided, and a *P*-value < 0.05 was considered statistically significant. All statistical analyses were performed using R version 4.1.3 in the R Studio environment [48].

## Results

**Fig. 2:**
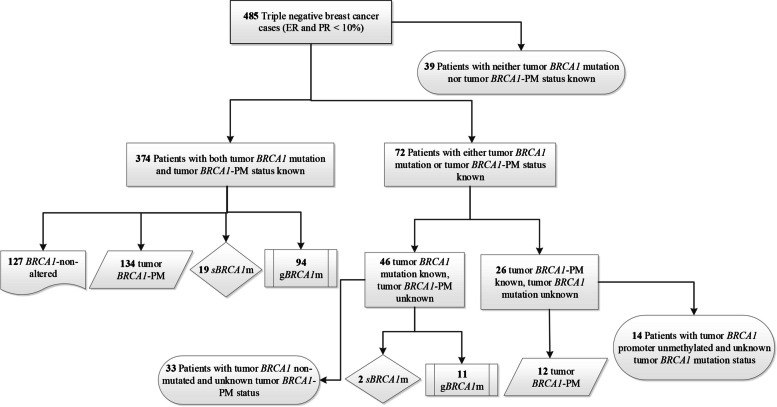
Classification of *BRCA1* mutation and tumor *BRCA1* promoter methylation. In total, 399 patients were classified into four groups: *BRCA1-*non-altered (*n* = 127), tumor *BRCA1*-PM (*n* = 134 + 12 = 146), s*BRCA1*m (*n* = 19 + 2 = 21), and g*BRCA1*m (*n* = 94 + 11 = 105). *Abbreviations*: ER, estrogen receptor; PR, progesterone receptor; *BRCA1*-PM, *BRCA1* promoter methylation; s*BRCA1*m, somatic *BRCA1* mutation; g*BRCA1*m, germline *BRCA1* mutation; *BRCA1*-non-altered, without germline *BRCA1* mutation, without somatic *BRCA1* mutation, and without tumor *BRCA1* promoter methylation

Of the 485 TNBC patients, 420 had valid results for both g*BRCA1*m and s*BRCA1*m status: 25.0% (105/420) carried a g*BRCA1*m, and 5.0% (21/420) carried only a s*BRCA1*m. Among the g*BRCA1*m carriers, one had an additional s*BRCA1*m and was considered as g*BRCA1*m in the analyses. We observed five patients with a germline *BRCA2* mutation, all of whom were g*BRCA1*wt. Details of the *BRCA1* mutations are shown in Additional file 3: Table S1. Tumor *BRCA1*-PM was present in 146 (36.5%) out of the 400 patients with available methylation status. Tumor *BRCA1* mutation (g*BRCA1*m or s*BRCA1*m) and tumor *BRCA1-*PM were mutually exclusive. Therefore, if a patient had a tumor *BRCA1* mutation and the methylation status was missing, we assumed the *BRCA1* promoter to be unmethylated, and vice versa. In total, 399 patients were classified into four groups: *BRCA1*-non-alteration (31.8%), g*BRCA1*m (26.3%), s*BRCA1*m (5.3%), and tumor *BRCA1*-PM (36.6%) (Fig. 2). *BRCA1*-like status, which was determined using tumors’ copy number profiles based on low-coverage whole-genome sequencing, was analyzed in 418 patients; 352 passed quality control, and 304 (86.4%) had *BRCA1*-like tumors. Most patients with a g*BRCA1*m (87.1%; 74/85), a s*BRCA1*m (82.4%; 14/17), or tumor *BRCA1*-PM (92.6%; 113/122) had *BRCA1*-like tumors. *BRCA1*-PM tumors had significantly lower *BRCA1* mRNA expression than g*BRCA1*m, s*BRCA1*m, or *BRCA1*-non-altered tumors (*P* < 0.001). We did not find any significant differences in sTILs, other clinicopathological characteristics, or treatment according to *BRCA1* status (Table 1) or *BRCA1*-like status (see Additional file 4: Table S2).

**Table 1:** Characteristics of all patients and patients with different *BRCA1* status

	**All patients**^**d**^** (*n* = 485)**	***BRCA1*-non-alteration (*n* = 127)**	**g*BRCA1*m (*n* = 105)**	**s*BRCA1*m (*n* = 21)**	**Tumor *BRCA1*-PM (*n* = 146)**	***P*-value**^**e**^
**Age at diagnosis, median (Q1–Q3), years**	35 (32–38)	35 (32–38)	35 (32–37)	35 (33–37)	35 (33–38)	0.545
**sTILs, median (Q1–Q3), %**	25 (5, 70)	23 (5, 65)	20 (10, 75)	27 (10, 65)	40 (5, 70)	0.448
Missing^a^	4	1	0	0	1	
**Tumor size, no. (%)**						
≤ 20 mm	285 (59.0)	69 (54.8)	66 (63.5)	9 (42.9)	87 (59.6)	0.268
> 20 mm	198 (41.0)	57 (45.2)	38 (36.5)	12 (57.1)	59 (40.4)	
Missing^a^	2	1	1	0	0	
**Tumor grade, no. (%)**						
Grade 1 or 2	70 (14.4)	17 (13.4)	12 (11.4)	2 (9.5)	14 (9.6)	0.795
Grade 3	415 (85.6)	110 (86.6)	93 (88.6)	19 (90.5)	132 (90.4)	
**Histological subtype, no. (%)**						
Carcinoma no special type	445 (91.8)	113 (89.0)	97 (92.4)	21 (100.0)	135 (92.5)	0.501
Metaplastic carcinoma	27 (5.6)	9 (7.1)	5 (4.8)	0 (0.0)	10 (6.8)	
Other subtypes	13 (2.7)	5 (3.9)	3 (2.9)	0 (0.0)	1 (0.7)	
**Lymphovascular invasion, no. (%)**						
No	429 (88.5)	110 (86.6)	94 (89.5)	17 (81.0)	131 (89.7)	0.550
Yes	56 (11.5)	17 (13.4)	11 (10.5)	4 (19.0)	15 (10.3)	
***BRCA1*-like tumor, no. (%)**						
Non-*BRCA1*-like	48 (13.6)	19 (19.2)	11 (12.9)	3 (17.6)	9 (7.4)	0.051
*BRCA1*-like	304 (86.4)	80 (80.8)	74 (87.1)	14 (82.4)	113 (92.6)	
Missing^a^	133	28	20	4	24	
***BRCA1* mRNA expression, median (Q1–Q3), normalized counts**	864.54 (273.81–1342.70)	1273.55 (905.61–1745.19)	1165.90 (864.00–1555.9)	911.90 (725.80–1366.7)	214.30 (132.26–320.54)	< 0.001
Missing^a^	133	23	19	5	36	
**Surgery type, no. (%)**						
Lumpectomy	324 (66.8)	86 (67.7)	62 (59.0)	12 (57.1)	102 (69.9)	0.333
Mastectomy	152 (31.3)	38 (29.9)	39 (37.1)	9 (42.9)	43 (29.5)	
Surgery not specified	9 (1.9)	3 (2.4)	4 (3.8)	0 (0.0)	1 (0.7)	
**Radiotherapy, no. (%)**						
No radiotherapy	141 (29.1)	36 (28.3)	35 (33.3)	8 (38.1)	39 (26.7)	0.550
Radiotherapy	344 (70.9)	91 (71.7)	70 (66.7)	13 (61.9)	107 (73.3)	
**Events of interest during 15-year follow-up**^**b**^**, no. (%)**						
Death due to any cause	137 (28.5)	37 (29.8)	40 (38.1)	7 (33.3)	32 (22.2)	NA
First distant recurrence	83 (17.3)	24 (19.4)	20 (19.0)	7 (33.3)	21 (14.6)	NA
Death without distant recurrence or second primary tumors	34 (7.1)	8 (6.5)	8 (7.6)	1 (4.8)	10 (6.9)	NA
First second primary tumors	85 (17.7)	17 (13.7)	48 (45.7)	1 (4.8)	9 (6.2)	NA
**The location of the first and second primary tumors**^**b**^**, no. (%)**						
Contralateral breast	64 (75.3)	13 (76.5)	40 (83.3)	1 (100)	5 (55.6)	NA
Ipsilateral breast	5 (5.9)	1 (5.9)	1 (2.1)	0 (0)	2 (22.2)	
Ovary	8 (9.4)	1 (5.9)	5 (10.4)	0 (0)	1 (11.1)	
Other locations^c^	8 (9.4)	2 (11.8)	2 (4.2)	0 (0)	1 (11.1)	
**Lost to follow-up, no. (%)**	8 (1.7)	1 (0.8)	1 (1.0)	1 (4.8)	3 (2.1)	NA

*Abbreviations*: *Q1* quartile 1, *Q3* quartile 3, *sTILs* stromal tumor-infiltrating lymphocytes, *BRCA1-non-alteration* without germline *BRCA1* mutation without somatic *BRCA1* mutation, and without tumor *BRCA1* promoter methylation, *gBRCA1m* germline *BRCA1* mutation, *sBRCA1m* somatic *BRCA1* mutation, *BRCA1-PM BRCA1* promoter methylation, *NA* not applicable

^a^Missing values were excluded when calculating the percentages and *P*-values

^b^Patients with a germline *BRCA2* mutation (*N* = 5) were excluded. These events were not mutually exclusive

^c^Other locations included the colon, lung, skin, and esophagus

^d^All patients included those without valid *BRCA1* status (*n* = 86)

^e^*P*-values were calculated using the Kruskal–Wallis tests, chi-square tests, or Fisher’s exact tests. Follow-up events were not compared across different *BRCA1* status; thus, no *P*-value was calculated

**Fig. 3:**
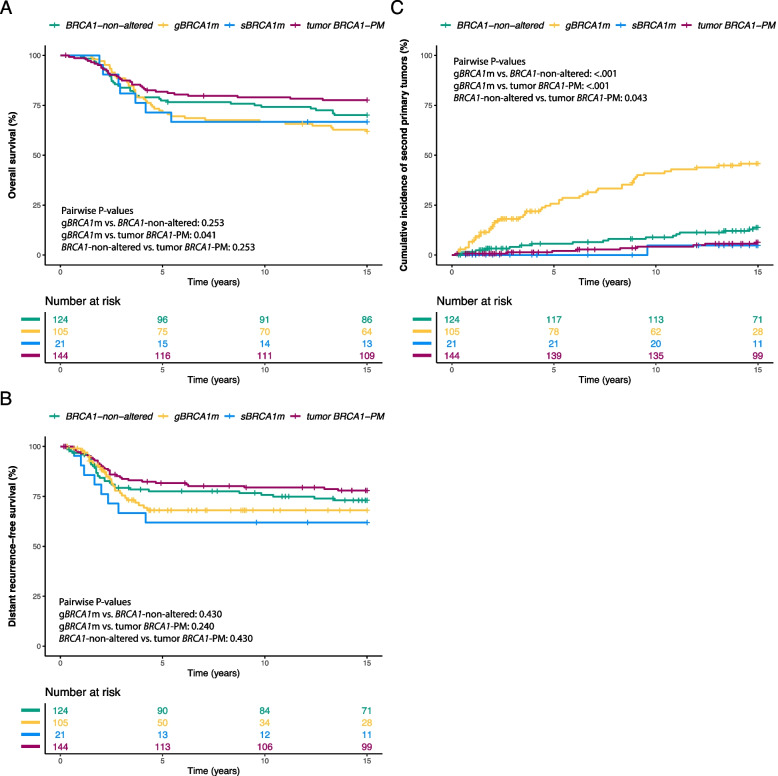
Clinical outcomes according to *BRCA1* status. Clinical outcomes include (**A**) overall survival, (**B**) distant recurrence-free survival, and (**C**) cumulative incidence of second primary tumors. Log-rank tests and Gray’s tests were used to compute the pairwise *P*-values. Comparison was only made among germline *BRCA1*-mutated, tumor *BRCA1* promoter-methylated, or *BRCA1*-non-altered patients, as the number of somatic *BRCA1*-mutated patients was too low. Pairwise *P*-values were corrected for multiple testing using the Benjamini–Hochberg procedure. The uncorrected *P*-values for overall survival comparison are as follows: g*BRCA1*m vs. *BRCA1-*non-altered (*P*-value = 0.253), g*BRCA1*m vs. tumor *BRCA1*-PM (*P*-value = 0.014), and tumor *BRCA1*-PM vs. *BRCA1-*non-altered (*P*-value = 0.189). The uncorrected *P*-values for distant recurrence-free survival comparison are as follows: g*BRCA1*m vs. *BRCA1-*non-altered (*P*-value = 0.429), g*BRCA1*m vs. tumor *BRCA1*-PM (*P*-value = 0.079), and tumor *BRCA1*-PM vs. *BRCA1-*non-altered (*P*-value = 0.328). The uncorrected *P*-values for the incidence of second primary tumors comparison are as follows: g*BRCA1*m vs. *BRCA1-*non-altered (*P*-value < 0.001), g*BRCA1*m vs. tumor *BRCA1*-PM (*P*-value < 0.001), and tumor *BRCA1*-PM vs. *BRCA1-*non-altered (*P*-value = 0.043). *Abbreviations*: *BRCA1*-non-altered, without germline *BRCA1* mutation, without somatic *BRCA1* mutation, and without tumor *BRCA1* promoter methylation; g*BRCA1*m, germline *BRCA1* mutation; s*BRCA1*m, somatic *BRCA1* mutation; tumor *BRCA1*-PM, tumor *BRCA1* promoter methylation. Note that at time 0, the numbers at risk of tumor *BRCA1* promoter methylated patients and *BRCA1*-non-altered patients were not 146 and 127, respectively, because five germline *BRCA2*-mutated patients were removed

During the 15-year follow-up, 137 patients died. Eighty-three patients first developed distant recurrence, 85 first developed second primary tumors, and 34 died without distant recurrence or second primary tumors. Eight patients were lost to the follow-up. Kaplan–Meier curves of OS and DRFS and cumulative incidence curves of second primary tumors stratified by *BRCA1* status are depicted in Fig. 3. Patients with g*BRCA1*m and tumor *BRCA1*-PM showed significantly different OS (Benjamini-Hochberg-corrected pairwise *P*-value = 0.041) and cumulative incidence of second primary tumors (Benjamini-Hochberg-corrected pairwise *P*-value < 0.001), although no statistically significant difference was observed in DRFS. The clinical outcomes at different follow-up times stratified by *BRCA1* status and sTIL levels are summarized in Table 2. Patients (*n* = 66) with tumor *BRCA1*-PM and sTILs ≥ 50% showed excellent 15-year OS (97.0%, 95% CI, 92.9–100%; Table 2), while patients (*n* = 61) with g*BRCA1*m and sTILs < 50% showed poor 15-year OS (50.8%; 95% CI, 39.7–65.0%; Table 2). The clinical outcomes stratified by *BRCA1* status and by *BRCA1*-like status are summarized in Additional file 5: Table S3 and Additional file 6: Table S4.

**Table 2:** Clinical outcomes according to different BRCA1 status and different levels of stromal tumor-infiltrating lymphocytes

	**No. of death**	**Overall survival (95% CI)**	**No. of distant recurrence or death**	**Distant recurrence-free survival (95% CI)**	**No. of second primary tumors**	**Cumulative incidence of second primary tumors (95% CI)**
**Tumor *BRCA1*-PM, sTILs < 50% (*n* = 77)**						
0 to 10 years	28	63.0 (53.0–74.9)	28	62.5 (52.4–74.5)	3	4.0 (0.0–8.3)
10 to 15 years	2	60.3 (50.2–72.4)	2	59.5 (49.3–71.9)	1	5.4 (0.1–10.3)
**Tumor *BRCA1*-PM, sTILs ≥ 50% (*n* = 66)**						
0 to 10 years	2	97.0 (92.9–100.0)	1	98.5 (95.6–100.0)	3	4.5 (0.0–9.4)
10 to 15 years	0	97.0 (92.9–100.0)	0	98.5 (95.6–100.0)	2	7.6 (1.0–13.8)
**g*BRCA1*m, sTILs < 50% (*n* = 61)**						
0 to 10 years	28	54.1 (42.9–68.2)	25	52.4 (40.3–68.0)	16	26.2 (15.0–36.0)
10 to 15 years	2	50.8 (39.7–65.0)	0	52.4 (40.3–68.0)	3	31.2 (19.5–41.2)
**g*BRCA1*m, sTILs ≥ 50% (*n* = 44)**						
0 to 10 years	7	84.1 (73.9–95.6)	3	91.4 (82.5–100.0)	27	61.4 (44.8–73.0)
10 to 15 years	3	77.1 (65.6–90.7)	0	91.4 (82.5–100.0)	2	65.9 (49.6–77.0)
**s*BRCA1*m, sTILs < 50% (*n* = 13)**						
0 to 10 years	6	53.8 (32.6–89.1)	7	46.2 (25.7–83.0)	0	0.0 (0.0–0.0)
10 to 15 years	0	53.8 (32.6–89.1)	0	46.2 (25.7–83.0)	0	0.0 (0.0–0.0)
**s*BRCA1*m, sTILs ≥ 50% (*n* = 8)**						
0 to 10 years	1	87.5 (67.3–100.0)	1	87.5 (67.3–100.0)	1	12.5 (0.0–32.7)
10 to 15 years	0	87.5 (67.3–100.0)	0	87.5 (67.3–100.0)	0	12.5 (0.0–32.7)
***BRCA1*-non-altered, sTILs < 50% (*n* = 77)**						
0 to 10 years	25	66.2 (56.3–77.9)	25	66.0 (56.1–77.8)	5	6.8 (0.9–12.3)
10 to 15 years	4	60.8 (50.6–73.0)	3	61.4 (51.1–73.8)	4	12.3 (4.6–19.4)
***BRCA1*-non-altered, sTILs ≥ 50% (*n* = 66)**						
0 to 10 years	7	85.7 (76.4–96.1)	4	91.0 (82.9–99.8)	6	12.2 (2.6–20.9)
10 to 15 years	1	83.6 (73.8–94.7)	0	91.0 (82.9–99.8)	2	16.3 (5.4–26.0)

*Abbreviations*: *tumor BRCA1-PM*, tumor *BRCA1* promoter methylation; *sTILs*, stromal tumor-infiltrating lymphocytes; *gBRCA1m*, germline *BRCA1* mutation; *BRCA1-non-alteration*, tumor without germline *BRCA1* mutation, somatic *BRCA1* mutation, or tumor *BRCA1* promoter methylation; *CI*, confidence interval

The multivariable Cox regression model showed that g*BRCA1*m patients had a worse OS from the fourth year after diagnosis compared to *BRCA1*-non-altered patients (HR_4–15 years_, 2.11; 95% CI, 1.18–3.75; Table 3). After additional adjustment for second primary tumors, the HR for g*BRCA1*m was attenuated (HR_4–15 years_, 1.43; 95% CI, 0.77–2.66). Patients with a s*BRCA1*m or tumor *BRCA1*-PM did not have significantly different OS compared to *BRCA1*-non-altered patients. Higher sTILs were associated with better OS (HR 0.84; 95% CI, 0.78–0.90) in g*BRCA1*m, s*BRCA1*m, or *BRCA1*-non-altered patients. This association was significantly larger in tumor *BRCA1*-PM patients, as was reflected by a significant interaction term between sTILs and tumor *BRCA1*-PM (HR_interaction_, 0.82; 95% CI, 0.68–0.98). This means that every 10% sTIL increment was associated with a 31% increase in OS for tumor *BRCA1*-PM patients and a 16% increase for patients with other *BRCA1* status. The HRs for other covariates are in Additional file 7: Table S5.

**Table 3:** (Subdistribution) hazard ratios for 15-year clinical outcomes according to *BRCA1* status, based on multiple-imputed data

	**OS, HR (95% CI)**	**OS with additional adjustment for second primary tumors**^**d**^**, HR (95% CI)**	**DRFS, HR (95% CI)**	**Second primary tumors**^**e**^**, sHR (95% CI)**
**Univariable**				
*BRCA1-*non-alteration	1.00 (referent)	NA	1.00 (referent)	1.00 (referent)
g*BRCA1*m 0–3 years^a^	0.73 (0.36–1.47)	NA	1.29 (0.79–2.11)	4.00 (2.34–6.86)
g*BRCA1*m 4–15 years^a^	2.00 (1.15–3.47)	NA		
s*BRCA1*m	1.17 (0.51–2.67)	NA	1.52 (0.67–3.44)	0.49 (0.07–3.41)
Tumor *BRCA1*-PM	0.72 (0.45–1.15)	NA	0.77 (0.47–1.27)	0.46 (0.21–1.02)
**Multivariable**^**b**^				
*BRCA1-*non-alteration	1.00 (referent)	1.00 (referent)	1.00 (referent)	1.00 (referent)
g*BRCA1*m 0–3 years^a^	0.75 (0.36–1.53)	0.60 (0.29–1.27)	1.34 (0.78–2.28)	4.04 (2.29–7.13)
g*BRCA1*m 4–15 years^a^	2.11 (1.18–3.75)	1.43 (0.77–2.66)		
s*BRCA1*m	0.96 (0.42–2.21)	1.02 (0.44–2.38)	1.30 (0.55–3.06)	0.49 (0.07–3.47)
Tumor *BRCA1*-PM	1.19 (0.65–2.16)	1.25 (0.68–2.28)	0.88 (0.51–1.51)	0.42 (0.19–0.95)
sTILs (every 10% increment)	0.84 (0.78–0.90)	0.82 (0.76–0.89)	0.74 (0.68–0.80)	1.11 (1.03–1.19)
sTILs by tumor *BRCA1*-PM status^c^	0.82 (0.68–0.98)	0.83 (0.69–1.00)	NA	NA

*Abbreviations*: *OS*, overall survival; *DRFS*, distant recurrence-free survival; *HR*, hazard ratio; *sHR*, subdistribution hazard ratio; *CI*, confidence interval; *BRCA1-non-alteration*, without germline *BRCA1* mutation, without somatic *BRCA1* mutation, and without tumor *BRCA1* promoter methylation; *gBRCA1m*, germline *BRCA1* mutation; *sBRCA1m*, somatic *BRCA1* mutation; *tumor BRCA1-PM*, tumor *BRCA1* promoter methylation; *sTILs*, stromal tumor-infiltrating lymphocytes; *NA*, not applicable

^a^Hazard ratios for germline *BRCA1* mutation were estimated for the first 3 years and from the fourth year onwards separately for overall survival because of non-proportional hazards

^b^Multivariable models were adjusted for stromal tumor-infiltrating lymphocytes (unit of 10%), tumor size (≤ 20 mm/ > 20 mm), tumor grade (grade 1 or 2/grade 3), histological subtype (carcinoma of no special type/metaplastic carcinoma/other subtypes), lymphovascular invasion (yes/no), and treatment (lumpectomy with radiotherapy/mastectomy alone/other treatments). Results of other covariates are summarized in Additional files 7–9: Table S5–S7

^c^For overall survival, a significant interaction term between stromal tumor-infiltrating lymphocytes (unit of 10%) and tumor *BRCA1* promoter methylation was added. Interaction terms between other *BRCA1* status and stromal tumor-infiltrating lymphocytes were not significant; thus, they were not included in the final model for overall survival. None of the interaction terms was significant in the models for distant recurrence-free survival or cumulative incidence of second primary tumors

^d^Second primary tumors (yes/no) was a time-varying covariate, i.e., with the value of 0 until the time when a second primary tumor occurred and with the value of 1 after that time

^e^Fine and Gray competing risk models were used to calculate subdistribution hazard ratios. Second primary tumors were the events of interest, and death and distant recurrence were competing events

Patients with g*BRCA1*m, s*BRCA1*m, or tumor *BRCA1*-PM did not have significantly different DRFS compared to *BRCA1*-non-altered patients (Table 3). Higher sTILs were associated with better DRFS in all patients. Although the interaction between sTILs and tumor *BRCA1*-PM was not statistically significant (the final model did not include this interaction term), the direction of the interaction was the same as in the model for OS. The HRs for other covariates are in Additional file 8: Table S6. Compared to *BRCA1-*non-altered patients, g*BRCA1*m patients had a higher incidence of second primary tumors (adjusted subdistribution HR, 4.04; 95% CI, 2.29–7.13; Table 3), while tumor *BRCA1-*PM patients had a lower incidence of second primary tumors (adjusted subdistribution HR, 0.42; 95% CI, 0.19–0.95; Table 3). There were no significant interaction terms between *BRCA1* status and sTILs for the incidence of second primary tumors. Subdistribution HRs and cause-specific HRs for *BRCA1* status (Additional file 9: Table S7 and Additional file 10: Table S8) were aligned.

Patients with *BRCA1*-like tumors did not have significantly different outcomes compared to patients with non-*BRCA1*-like tumors (Additional file 11: Table S9). Results from the sensitivity analyses (Additional file 7–10: Table S5–S8) aligned with the results from the main analysis. Results of the complete-case analysis (Additional file 12: Table S10) also aligned with the results using multiple-imputed data.

## Discussion

In this population-based cohort of young, node-negative TNBC patients, we compared patients’ clinical outcomes independent of the curative effect of adjuvant chemotherapy across different *BRCA1* status and *BRCA1*-like status. In addition, we investigated the prognostic value of sTILs in patients with different *BRCA1* status and identified subgroups of patients with distinct risks. These findings have the potential to improve risk classification in young, node-negative TNBC patients.

Our study found that g*BRCA1*m was associated with worse OS in young, node-negative TNBC patients, consistent with several previous studies predominantly involving chemotherapy-naïve patients [23, 49, 50]. However, more recent data, including mainly chemotherapy-treated patients with or without risk-reducing surgeries, showed that germline *BRCA1*/*2* mutations did not negatively impact the survival of TNBC patients [5, 6, 24, 25, 51–54]. When combined with the results of previous studies, our findings suggest that chemotherapy could considerably improve the OS of g*BRCA1*m patients.

Furthermore, we showed that young TNBC patients with a g*BRCA1*m had a significantly increased risk of second primary tumors, primarily contralateral breast tumors, which is consistent with a recent prospective cohort study [55]. Given that these second primary tumors contributed significantly to worse OS in our study population, it is necessary to consider risk-reducing surgery for young, node-negative TNBC patients who carry a g*BRCA1*m. However, it is important to note that the negative impact of second primary tumors on OS should not raise unnecessary anxiety to give risk-reducing surgery to young TNBC patients who have no genetic or familial risk factors [56]. We showed a relatively low incidence of second primary tumors in g*BRCA1*wt patients, especially in tumor *BRCA1*-PM patients. The incidence may have been lower after chemotherapy, as was shown by previous studies that chemotherapy reduces the risk of contralateral breast cancers [57–59]. Therefore, risk-reducing surgery should, in line with most guidelines, only be offered to patients with a predicted high risk of second primary tumors [60]. Nevertheless, our results, derived from this unique chemotherapy-naïve cohort with young, node-negative TNBC patients, can facilitate transparent risk communication and a shared treatment decision-making between oncologists and patients.

Results on the prognostic value of tumor *BRCA1*-PM in TNBC patients have been conflicting [26, 61–65], which may be due to different methods to analyze *BRCA1*-PM status [66, 67], different reference groups (including g*BRCA1*m patients or not), or different treatments [62, 64]. Our study found no significant difference in OS or DRFS between patients with tumor *BRCA1*-PM and *BRCA1*-non-altered patients. Interestingly, we found that tumor *BRCA1*-PM may modify the association between sTILs and OS, as shown by a nearly two-fold increase in OS for tumor *BRCA1*-PM patients with every 10% increment of sTILs, compared to those with other *BRCA1* status. Combined with the result of the similar distribution of sTILs across the *BRCA1* status, this stronger association suggests that sTIL compositions or spatial relationships with the tumor cells might differ between patients with and without tumor *BRCA1*-PM. Future research may consider using a comprehensive technique such as imaging mass cytometry [68] to compare the sTIL compositions and spatial relationships among TNBCs with different *BRCA1* status.

Our previous study has shown that patients without tumor *BRCA1* mutation and high sTILs may have the potential to forgo chemotherapy [16]. With further information on g*BRCA1*m and tumor *BRCA1*-PM, we redid the risk classification, and two distinct subgroups were identified. One group, characterized by high sTILs and tumor *BRCA1*-PM, showed excellent 15-year OS and DRFS, while the other group, characterized by low sTILs and g*BRCA1*m showed poor 15-year OS and DRFS. These results, once validated, have the potential to aid adjuvant treatment intensification and optimization in young, node-negative TNBC patients.

We found a lower incidence of second primary tumors in tumor *BRCA1*-PM patients, compared to *BRCA1*-non-altered patients. To date, we have no biological explanation for this novel association, and it might have been a chance finding. Preliminary analysis using DNA from tumor-free lymph nodes of 19 tumor *BRCA1*-PM patients showed no (constitutional) methylation of *BRCA1*. The association might have been overestimated due to the potential misclassification of g*BRCA1*m patients as *BRCA1*-non-altered, resulting in a higher incidence of second primary tumors in the *BRCA1*-non-altered group. However, the mutual exclusiveness between g*BRCA1*m and *BRCA1*-PM, which has been reported in many studies [22, 69–71], minimized the chance of misclassifying g*BRCA1*m patients into the *BRCA1*-PM group. Therefore, if validated, it would be interesting to further consider the clinical relevance of testing *BRCA1*-PM in young, node-negative TNBC patients.

The prevalence of g*BRCA1*m, s*BRCA1*m, and tumor *BRCA1*-PM in our cohort was similar to previous studies [6, 22, 26, 71, 72]. In addition, our study showed that young TNBC patients predominantly had *BRCA1*-like tumors, which aligns with other studies [22, 26, 73], regardless of different homologous recombination deficiency (HRD) classifiers being used. Although our study did not cross-validate tumors’ *BRCA1*-like status using other genomic measures, a recent study reported a 70% concordance between the *BRCA*-like classifier and the functional DNA repair capacity assays (RECAP), as well as the whole-genome sequencing-based Classifier of HOmologous Recombination Deficiency (CHORD) assay [74]. In addition, this study showed that *BRCA*-like tumors are enriched for tumor mutational signature 3 [74]. In our study, most tumors with g*BRCA1*m, s*BRCA1*m, or *BRCA1*-PM were classified as *BRCA1*-like, while only a small proportion were classified as non-*BRCA1*-like. These non-*BRCA1*-like tumors may have arisen sporadically, as was reported that the absence of locus-specific loss of heterozygosity was observed in 10% of g*BRCA1*m breast tumors and their HRD scores were similar to sporadic tumors [75]. However, our sensitivity analysis focusing only on patients with *BRCA1*-like tumors yielded similar results to the main analysis that included all patients. Moreover, the *BRCA1*-like classifier, as many other HRD classifiers, is not 100% accurate for detecting *BRCA1*-altered tumors [22, 26].

This study had several unique strengths. First, indication bias was minimized because all chemotherapy-naïve patients were treated according to the guidelines in the specific era of diagnosis. Including TNBC patients from a more recent era might lead to an underestimation of the negative impact of g*BRCA1*m, since currently, only those with an extremely low risk might forgo chemotherapy [76]. Second, immortal time bias was not an issue in this study since *BRCA1* status was tested using archived tissues. Studies including prevalent patients who had to survive to be tested might have underestimated the effect of g*BRCA1*m. Third, the g*BRCA1*m patients in our study were unlikely to receive prophylactic mastectomy and salpingo-oophorectomy due to the lack of awareness of their mutation status at diagnosis. Although we lacked information on prophylactic surgery and subsequent surgery after the diagnosis of TNBC, *BRCA1* mutation was only discovered in 1994, and genetic testing was not introduced in the Netherlands until 1995, followed by its implementation in the clinic.

Our study may not have been completely free of bias. One potential source of bias is that young patients with family histories may have been referred to clinical genetics after 1995, and those who were found to carry a g*BRCA1*m might have chosen risk-reducing treatments that could have improved their outcomes. However, g*BRCA1*m carriers were more likely to receive chemotherapy [50] and were excluded from our cohort, which may have partially counterbalanced such an impact on our findings. Besides, our study only focused on *BRCA1* mutations, whereas other gene mutations associated with TNBC, such as *BRCA2*, *RAD51C/D*, *BARD1*, and *PALB2* [8, 77], might also have influenced the outcomes. Nevertheless, the proportion of other germline mutations in young TNBC patients is very low [8]. Lastly, all patients were of European descent; thus, generalization to other ethnicities should be made carefully.

## Conclusions

In conclusion, although both g*BRCA1*m and tumor *BRCA1*-PM alter *BRCA1* gene transcription, they were associated with significantly different outcomes in young, node-negative TNBC patients. The prognostic value of sTILs remained across patients with different *BRCA1* status, albeit this association was stronger in those with tumor *BRCA1*-PM. Combining sTILs and *BRCA1* status has the potential to improve risk classification and tailored adjuvant treatment in this patient population. Furthermore, the high incidence of second primary tumors in young g*BRCA1*m carriers and its association with worse OS emphasize the importance of risk-reducing surgery or active monitoring. Such decisions should be discussed between physicians and patients with transparent information being provided, taking family planning into account [60].

### Supplementary Information

**Additional file 1.** Supplementary Methods.

**Additional file 2: Fig. S1.** Flow chart of tumor *BRCA1* mutation testing.

**Additional file 3: Table S1.** Locations of the germline and somatic *BRCA1* mutations.

**Additional file 4: Table S2.** Clinicopathological characteristics, *BRCA1* mRNA expression, treatment, and follow-up events of patients with non-* BRCA1*-like or *BRCA1*-like tumors.

**Additional file 5: Table S3.** 3-, 5-, 10-, and 15-year overall survival rate, distant recurrence-free survival rate, and cumulative incidence of second primary tumors according to *BRCA1* status.

**Additional file 6: Table S4.** 3-, 5-, 10-, and 15-year overall survival rate, distant recurrence-free survival rate, and cumulative incidence of second primary tumors according to *BRCA1*-like status.

**Additional file 7: Table S5.** Hazard ratios for overall survival according to *BRCA1* status, based on multiple-imputed data.

**Additional file 8: Table S6.** Hazard ratios for distant recurrence-free survival according to *BRCA1* status, based on multiple-imputed data.

**Additional file 9: Table S7.** Subdistribution hazard ratios for second primary tumors according to *BRCA1* status, based on multiple-imputed data, using Fine and Gray competing risk models with distant recurrence and death as competing events.

**Additional file 10: Table S8.** Hazard ratios for second primary tumors according to *BRCA1* status, based on multiple-imputed data, using cause-specific competing risk models with distant recurrence and death as competing events.

**Additional file 11.** Univariable (subdistribution) hazard ratios according to *BRCA1*-like status.

**Additional file 12.** (subdistribution) Hazard ratios according to *BRCA1* status, based on cases with complete information.

## Data Availability

The clinical data in this study are available from the Netherlands Cancer Registry, hosted by the Netherlands Comprehensive Cancer Organization; however, restrictions apply to the availability of these data, which were used under license for the current study. Other data generated (*BRCA1*-related variables, sTILs) are available from the authors upon reasonable request and with permission from the Netherlands Comprehensive Cancer Organization.

## Abbreviations

*BRCA1* non-alteration: Without germline *BRCA1* mutation, without somatic *BRCA1* mutation, and without tumor *BRCA1* promoter methylation
*BRCA1*-PM: *BRCA1* Promoter methylation
DRFS: Distant recurrence-free survival
g*BRCA1*m: Germline *BRCA1* mutation
g*BRCA1*wt: Germline *BRCA1* wild-type
HR: Hazard ratio
HRD: Homologous recombination deficiency
indels: Insertions or deletions
NKI: Netherlands Cancer Institute
OS: Overall survival
s*BRCA1*m: Somatic *BRCA1* mutation
SNVs: Single-nucleotide variants
sTILs: Stromal tumor-infiltrating lymphocytes
TILs: Tumor-infiltrating lymphocytes
TNBC: Triple-negative breast cancer
