# Supplementary material for: Long-term outcomes of young, node-negative, chemotherapy-naïve, triple-negative breast cancer patients according to BRCA1 status
Source: BMC Med. 2024 Jan 9;22:9. doi: 10.1186/s12916-023-03233-7 (PMC10775514; doi:10.1186/s12916-023-03233-7)
Supplement: Supplementary file 1 — Additional file 1. Supplementary Methods. [file 12916_2023_3233_MOESM1_ESM.docx]

# **Supplementary Methods**

### **Breast cancer subtype classification and pathological characteristics review**

For the PARADIGM study, we requested formalin-fixed, paraffin-embedded tumor blocks with individual pathology reports through PALGA, the nationwide network, and registry of histology and cytopathology in the Netherlands. Fresh slides were cut and stained with hematoxylin and eosin. Specialized breast pathologists revised all available tissue specimens, blinded to follow-up information and clinicopathological records. Tissue microarrays were constructed and stained for estrogen receptor (ER), progesterone receptor (PR) and human epidermal growth factor receptor 2 (HER2). In addition, silver in situ hybridization (SISH) for HER2 was performed (details available upon request). Tumors with ER and PR expression < 10% were considered as ER- and PR-negative. For HER2 status, immunohistochemistry 0/1, or immunohistochemistry 2+ and SISH-negative/equivocal were considered HER2-negative. In this study, we included all patients with confirmed ER-negative, PR-negative and HER2-negative tumors (n = 485).

### **DNA and RNA isolation**

Tumor DNA, RNA and normal DNA was isolated in the Core Facility of Molecular Pathology & Biobanking, the NKI. A pathologist indicated the most tumor-dense region for tumor DNA and RNA isolation on hematoxylin and eosin-stained slides and scored tumor percentage in the marked area. For DNA and RNA isolation, 5 to 10 (depending on the tumor size) slides (10 µm thickness) from formalin-fixed paraffin-embedded (FFPE) tumor tissues were used. Tumor DNA and RNA was isolated simultaneously with the AllPrep DNA/RNA FFPE isolation kit (Qiagen, catalog nr. #80234) by using the QIAcube, according to manufacturer’s protocol. Normal DNA was isolated from unaffected FFPE lymph nodes if available, otherwise from normal breast tissue, using QiaAmp DNA FFPE Tissue kit (Qiagen, catalog nr. #56404) according to manufacturer’s protocols. Qubit dsDNA high sensitivity kit was used to measure the concentration of the double-stranded DNA. NanoDrop was used for RNA concentration measurement.

### **Pathogenic germline/somatic *BRCA1/2* mutation and tumor *BRCA1* promoter methylation**

We sent 120 ng of double-stranded tumor DNA per patient from in total 455 patients for *BRCA1/2* sequencing to Multiplicom (Niel, Belgium), which has been incorporated into Agilent (Carpinteria, CA, USA), using SureSelect and SureMASTR HRR kits according to manufacturer’s protocol. DNA was analyzed for single-nucleotide variants, and small insertions or deletions in the *BRCA1/2* genes. Due to the age of our tissue samples, extracted DNA was degraded, which led to high numbers of artifact variants in some samples and even failures of variants detection. Of the 455 tumor DNA samples, vcf files of 417 samples were successfully generated by Agilent, and analyzed in Bench Lab NGS v4.3.5 (Agilent Technologies) by an expert clinical molecular geneticist in the NKI. *BRCA1/2* variants were assessed according to diagnostic standard. In general, variants that result in a premature termination codon (stop-gain), a frameshift or disrupted canonical splicesite (-1/2 or +1/2) were considered as (likely) pathogenic mutations. Non-synonymous variants were assessed by weighing national and international expert classification, ACMG rules and literature. Tumor *BRCA1/2* mutations were confirmed using Sanger sequencing with 30 ng total tumor DNA per patient, and then analyzed using Sanger sequencing with 30 ng total DNA per patient from matched normal tissue.

A maximum of 80 ng of double-stranded tumor DNA per patient was used to determine tumor *BRCA1* promoter methylation using methylation-specific multiplex ligation-dependent probe amplification (MS-MLPA). MLPA reagents (panel ME053-X1, not commercially available) were obtained from MRC-Holland, Amsterdam, the Netherlands. Panel ME053-X1 contains three probes to detect three cytosine phosphate guanine (CpG) sites in the *BRCA1* promoter region. Each probe contains a HhaI (methylation-sensitive endonuclease) recognition site and can target CpG dinucleotide within a CpG island [32]. If the CpG island was unmethylated, HhaI would digest the DNA-MS-MLPA probe complex and no amplification product would be formed and therefore produced no signal. If the CpG island was methylated, the DNA-MS-MLPA probe complex would be amplified during subsequent PCR cycles and therefore a normal signal would be generated. The methylation percentage was calculated by comparing the probe signals of the HhaI-treated and -untreated reactions. A sample was determined to be tumor *BRCA1* promoter-methylated when three CpG islands were methylated. If only two CpG islands were methylated, the sample would be tested again using ME001-D1 probeset, which is used in clinical diagnosis, for a final decision. In addition, a randomly selected subset of both *BRCA1* promoter methylated and unmethylated samples were repeated with the commercially available ME001-D1 probeset, of which the results remained the same as using ME053-X1.

Since it was challenging to detect large deletions in the *BRCA1* gene using SureSelect and SureMASTR HRR kits (Agilent Technologies), we tested two Dutch founder mutations using deletion specific PCR in: (1) tumors without a *BRCA1* mutation and without *BRCA1* promoter methylation (N=146); (2) tumors without a *BRCA1* mutation and with an unknown status of *BRCA1* promoter methylation (n=34); (3) tumors with *BRCA1* promoter methylation and an unknown status of *BRCA1* mutation (n=15); (4) tumors with unknown status of both *BRCA1* mutation and *BRCA1* promoter methylation (n=41). The two mutations were *BRCA1* exon 13 (c.4186-1632_4357+2031del) and exon 22 (c.5333-36_5406+400del) deletions, which are prevalent in the Dutch population [33].

### **Low coverage whole genome sequencing and *BRCA1*-like status**

A *BRCA1*-like classifier developed previously was used to distinguish *BRCA1*-like and non-*BRCA1*-like copy number profiles. This classifier is a shrunken centroids classifier originally developed using array comparative genomic hybridization data from germline *BRCA1-*mutated and sporadic breast cancer patients [12]. It results in a probability score ranging from 0 to 1, describing how much a tumor copy number profile resembles a typical *BRCA1*-mutated tumor profile or a sporadic tumor profile. The copy number profiles obtained using low coverage whole genome sequencing can be reliably used for *BRCA1*-like classification [34, 35] and the threshold for the classification as *BRCA1*-like (≥ 0.63) or non-*BRCA1*-like was previously established and validated [36]. Tumor DNA was isolated as described above. Each tumor DNA sample was required to have at least 40% tumor cell percentage and 100ng of double-stranded DNA. Copy number profiles were obtained using low coverage whole genome sequencing as described before [35]. Samples with low quality were excluded from analyses.

### ***BRCA1* mRNA expression**

Tumor mRNA (250ng per sample based on NanoDrop) was sequenced in the Genomics Core Facility, the NKI. mRNA sequencing data was available for 463 patients, 352 of whom passed the quality control. The sequencing data were aligned to the reference genome GRCh38 with STAR (version 2.7.1a) [37] with the two-pass mode option set to “Basic”, and gene counts were obtained using STAR-quantMode = GeneCounts option. Subsequently, median of ratios normalization was performed with Deseq2 R package (version 1.23.0) [38].

### **Statistical analysis**

#### **Non-proportional hazard ratios**

Overall survival curves, distant recurrence-free survival curves, and cumulative incidence curves of second primary tumors were derived from cases with complete information. We examined proportional hazards assumptions using multiple-imputed data for overall survival, distant recurrence-free survival, and cumulative incidence of second primary tumors. Only the hazard ratio for g*BRCA1*m on overall survival was not proportional over time, and the plot of Schoenfeld residual indicated a change in the relationship between the residuals and follow-up time at around 3 years after diagnosis. Therefore, the hazard ratio for g*BRCA1*m was estimated for the first three years (HR_0-3 years_) and from the fourth year onwards (HR_4-15 years_) separately.

#### **Covariates and multicollinearity**

In order to adjust the effect of *BRCA1* status for potential mediators or confounders, we included tumor characteristics and treatment information in multivariable regression models. The included covariates were tumor size (> 20mm versus ≤ 20mm), tumor grade (grade 3 versus grade 1 and 2), stromal tumor infiltrating lymphocytes (continuous), lymphovascular invasion (yes versus no), histological subtypes (metaplastic carcinoma versus carcinoma no special type; other types versus carcinoma no special type), and locoregional treatment of the first breast cancer (mastectomy versus lumpectomy with radiotherapy; other treatment (i.e. mastectomy with radiotherapy or lumpectomy alone, or unknown surgery types) versus lumpectomy with radiotherapy). We did not include age as a covariate because the age range in our cohort was relatively narrow (patients were between 22 and 39 years of age at the time of diagnosis) and including age in the models did not cause a more than 10% of change of the unadjusted effects of *BRCA1* status, i.e., age was not a confounder. Multicollinearity was examined in each multivariable model using the variance inflation factor in the complete-case analysis. The variance inflation factors of all covariates were under two, indicating there was no obvious multicollinearity in the multivariable models.

#### **Multiple imputation**

We assumed that missing values in *BRCA1* status were missing at random. This assumption was based on the observation in our data that tumors with lower grade, smaller size or special histological types were significantly more likely to have missing information on tumor *BRCA1* mutation, tumor *BRCA1* promoter methylation and *BRCA1*-like status. Missing values in germline/somatic *BRCA1* mutation (N = 65), tumor *BRCA1* promoter methylation (N = 85), *BRCA1*-like status (N= 133), continuous tumor size (N=57, note that categorical tumor size was only missing in two patients, as was reported in Table 1), and stromal tumor infiltrating lymphocytes (N=4) were imputed using multiple imputation by chained equations (MICE package version 3.15.0). Missing values of germline and somatic *BRCA1* mutation, and *BRCA1*-like status were imputed using logistic regression models for binary variable. Missing values of tumor *BRCA1* promoter methylation were imputed using logistic regression models for binary variable and were conditionally on the complete and imputed values of germline and somatic *BRCA1* mutations, i.e. if a germline or somatic *BRCA1* mutation was positive, tumor *BRCA1* promoter methylation was imputed as negative. Missing values in tumor size and stromal tumor infiltrating lymphocytes were imputed with a predictive mean matching for continuous variable. All covariates with complete information were included as predictors in the imputation models. We also included auxiliary variables such as incidence year (from 1989 to 2000, continuous variable), pushing border (absent/ present in < 25% of the tumor perimeter/ present in 25%-75% of the tumor perimeter/ present in > 75% of the tumor perimeter), fibrotic focus (from 0 to 17, continuous variable), central necrosis (absent/ present), and tumor *BRCA2* mutation (tumor *BRCA2* wild type/ germline *BRCA2* mutation/ somatic *BRCA2* mutation). Moreover, separate imputation models were created for the different clinical outcomes. Information on the clinical outcomes was included in the imputation model using occurrence of the outcome as a binary variable yes/no and baseline cumulative hazard obtained with the Nelson-Aalen estimator or baseline cumulative subhazards obtained with Nelson-Johansen estimator. We imputed 20 datasets using 25 iterations. Cox regression models and competing risk models were then built using data from each imputed data set and the results were pooled using the pool function (based on Rubin’s rule) from the MICE package.
