# Supplementary material for: Long-term outcomes of young, node-negative, chemotherapy-naïve, triple-negative breast cancer patients according to BRCA1 status
Source: BMC Med. 2024 Jan 9;22:9. doi: 10.1186/s12916-023-03233-7 (PMC10775514; doi:10.1186/s12916-023-03233-7)
Supplement: Supplementary file 2 — Additional file 2: Fig. S1. Flow chart of tumor BRCA1 mutation testing. [file 12916_2023_3233_MOESM2_ESM.docx]

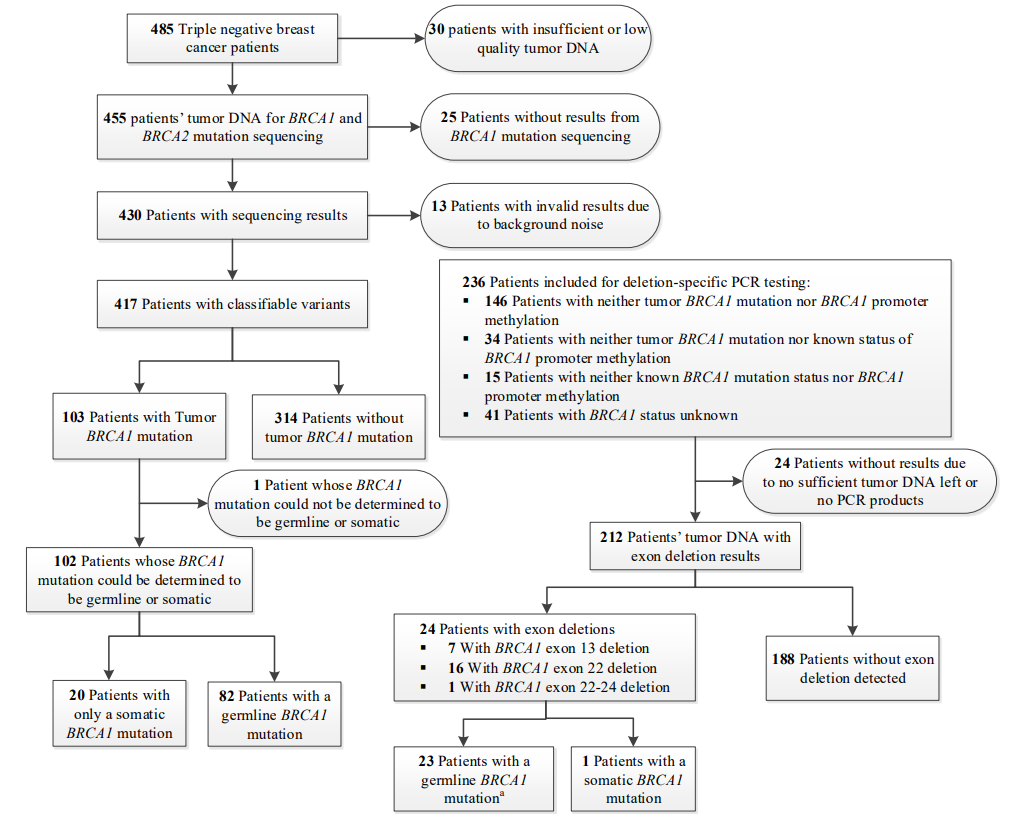


**Figure S1. Flow chart of tumor *BRCA1* mutation testing**

^a^ Among the 23 cases with germline *BRCA1* exon deletion, 3 were failed to be confirmed due to no germline tissue/DNA available. An expert geneticist suggested to treat them as germline, since these are recognized as common germline mutations.
