# Supplementary material for: Long-term outcomes of young, node-negative, chemotherapy-naïve, triple-negative breast cancer patients according to BRCA1 status
Source: BMC Med. 2024 Jan 9;22:9. doi: 10.1186/s12916-023-03233-7 (PMC10775514; doi:10.1186/s12916-023-03233-7)
Supplement: Supplementary file 3 — Additional file 3: Table S1. Locations of the germline and somatic BRCA1 mutations. [file 12916_2023_3233_MOESM3_ESM.docx]

## **Table S1. Locations of the germline and somatic BRCA1 mutations**

| **Germline (likely) pathogenic variants** | |
| --- | --- |
| **Frameshift** | **Frequency** |
| c.66dup p.Glu23fs | 4 |
| c.68_69del p.Glu23fs | 3 |
| c.697_698del p.Val233fs | 1 |
| c.1287dup p.Asp430fs | 1 |
| c.1292dup p.Leu431fs | 2 |
| c.1961del p.Lys654fs | 1 |
| c.2019del p.Glu673fs | 2 |
| c.2197_2201del p.Glu733fs | 6 |
| c.2212_2215del p.Val738fs | 1 |
| c.2685_2686del p.Pro897fs | 8 |
| c.2694dup p.Val899fs | 1 |
| c.3329dup p.Gln1111fs | 1 |
| c.3485del p.Asp1162fs | 2 |
| c.3616del p.Ala1206fs | 1 |
| c.3710del p.Ile1237fs | 1 |
| c.3770_3771del p.Glu1257fs | 2 |
| c.3820dup p.Val1274fs | 2 |
| c.4146_4155dup p.Ser1386fs | 1 |
| c.4391_4393delCTAinsTT p.Pro1464fs | 1 |
| c.4483del p.Arg1495fs | 1 |
| c.5030_5033del p.Thr1677fs | 1 |
| c.5177_5180del p.Arg1726fs | 1 |
| c.5266dup p.Gln1756fs | 4 |
| Sum | 48 |
| **Splice** | **Frequency** |
| c.302-3C>G p.? | 1 |
| c.5277+1G>A p.? | 12 |
| Sum | 13 |
| **Missense** | **Frequency** |
| c.5095C>T p.Arg1699Trp | 1 |
| c.5509T>C p.Trp1837Arg | 1 |
| Sum | 2 |
| **Nonsense** | **Frequency** |
| c.1687C>T p.Gln563* | 1 |
| c.2338C>T p.Gln780* | 3 |
| c.3748G>T p.Glu1250* | 4 |
| c.4165_4166del p.Ser1389* | 2 |
| c.5080G>T p.Glu1694* | 2 |
| c.5251C>T p.Arg1751* | 1 |
| c.5346G>A p.Trp1782* | 1 |
| c.5353C>T p.Gln1785* | 1 |
| c.5503C>T p.Arg1835* | 4 |
| Sum | 19 |

(Continued)

| **Somatic (likely) pathogenic variants** | |
| --- | --- |
| **Exon deletions** | **Frequency** |
| Exon 13 | 7 |
| Exon 22 | 16 |
| Sum | 23 |
| **Frameshift** | **Frequency** |
| c.126_127dup p.Phe43fs | 1 |
| c.415_416del p.Gln139fs | 1 |
| c.875del p.Leu292fs | 1 |
| c.1016dup p.Val340fs | 1 |
| c.1086_1087del p.Asn363fs | 1 |
| c.1729_1730del p.Glu577fs | 1 |
| c.1787_1803del p.Leu596fs | 1 |
| c.1961del p.Lys654fs | 1 |
| c.2685_2686del p.Pro897fs | 1 |
| Sum | 9 |
| **Splice** | **Frequency** |
| c.80+1G>A p.? | 1 |
| c.302-1G>A p.? | 1 |
| c.5332+1G>A | 1 |
| Sum | 3 |
| **Missense** | **Frequency** |
| c.191G>A p.Cys64Tyr | 1 |
| Sum | 1 |
| **Nonsense** | **Frequency** |
| c.604C>T p.Gln202* ^a^ | 1 |
| c.2410C>T p.Gln804* | 1 |
| c.3122C>G p.Ser1041* | 1 |
| c.4327C>T p.Arg1443* | 2 |
| c.5503C>T p.Arg1835* | 2 |
| c.5390C>A p.Ser1797* | 1 |
| Sum | 8 |
| **Exon deletions** | **Frequency** |
| Exon 22 to 24 | 1 |
| Sum | 1 |

^a^ This mutation co-occurred with a germline *BRCA1* mutation c.2685_2686delAA p.Pro897fs
