# Supplementary material for: Long-term outcomes of young, node-negative, chemotherapy-naïve, triple-negative breast cancer patients according to BRCA1 status
Source: BMC Med. 2024 Jan 9;22:9. doi: 10.1186/s12916-023-03233-7 (PMC10775514; doi:10.1186/s12916-023-03233-7)
Supplement: Supplementary file 4 — Additional file 4: Table S2. Clinicopathological characteristics, BRCA1 mRNA expression, treatment, and follow-up events of patients with non- BRCA1-like or BRCA1-like tumors. [file 12916_2023_3233_MOESM4_ESM.docx]

## **Table S2. Clinicopathological characteristics, *BRCA1* mRNA expression, treatment, and follow-up events of patients with non-*BRCA1*-like or *BRCA1*-like tumors**

|  | **No. (%)** | | |
| --- | --- | --- | --- |
|  | **Non-*BRCA1*-like**  **(n = 48)** | ***BRCA1*-like**  **(n = 304)** | ***P*-value ^d^** |
| **Age at diagnosis, median (Q1-Q3), years** | 34 (32-37) | 35 (32-38) | 0.307 |
| **sTILs, median (Q1-Q3), %** | 55 (10-75) | 20 (5-70) | 0.059 |
| Missing ^a^ | 0 | 1 |  |
| **Tumor size, No. (%)** | | | |
| ≤20mm | 31 (64.6) | 163 (54.0) | 0.223 |
| >20mm | 17 (35.4) | 139 (46.0) |  |
| Missing ^a^ | 0 | 2 |  |
| **Tumor grade, No. (%)** | | | |
| Grade 1 or 2 | 6 (12.5) | 35 (11.5) | >.99 |
| Grade 3 | 42 (87.5) | 269 (88.5) |  |
| **Histological subtype, No. (%)** | | | |
| Carcinoma no special type | 41 (85.4) | 285 (93.8) | 0.069 |
| Metaplastic carcinoma | 7 (14.6) | 17 (5.6) |  |
| Other subtypes | 0 (0) | 2 (0.7) |  |
| **Lymphovascular invasion, No. (%)** | | | |
| No | 41 (85.4) | 275 (90.5) | 0.304 |
| Yes | 7 (14.6) | 29 (9.5) |  |
| ***BRCA1* mRNA expression, median (Q1-Q3), normalized counts** | 924.20  (479.60-1247.90) | 857.92  (230.53-1305.01) | 0.575 |
| Missing | 10 | 68 |  |
| **Surgery types, No. (%)** | | | |
| Lumpectomy | 27 (56.2) | 204 (67.1) | 0.128 |
| Mastectomy | 19 (39.6) | 96 (31.6) |  |
| Surgery not specified | 2 (4.2) | 4 (1.3) |  |
| **Radiotherapy, No. (%)** | | | |
| No radiotherapy | 20 (41.7) | 87 (28.6) | 0.097 |
| Radiotherapy | 28 (58.3) | 217 (71.4) |  |
| **Events of interest during 15-year follow-up ^b^, No. (%)** | | | |
| Death due to any cause | 15 (31.2) | 84 (28.1) | NA |
| First distant recurrence | 9 (18.8) | 51(17.1) |  |
| Death without distant recurrence or second primary tumors | 3 (6.2) | 24 (8.0) |  |
| First second primary tumors | 11 (22.9) | 52 (17.4) |  |
| **The location of first second primary tumors ^b^, No. (%)** | | | |
| Contralateral breast | 8 (72.7) | 40 (76.9) | NA |
| Ipsilateral breast | 0 (0) | 4 (7.7) |  |
| Ovary | 2 (18.2) | 6 (11.5) |  |
| Other locations ^c^ | 1 (9.1) | 2 (3.8) |  |
| **Lost to follow-up, No. (%)** | 1 (2.1) | 3 (1.0) |  |

Abbreviations: Q1, quartile 1; Q3, quartile 3; sTILs, stromal tumor infiltrating lymphocytes; NA, not applicable.

^a^ Percentages of each clinicopathological characteristics were calculated excluding missing data.

^b^ Patients with a confirmed germline *BRCA2* mutation (n = 5) were excluded.

^c^ Other locations included colon, lung, skin, and esophagus.

^d^ *P*-values were calculated using Kruskal-Wallis tests, Chi-square tests or Fisher’s exact tests. Follow-up events were not compared across different *BRCA1*-like status, thus no *P*-value was calculated.
