# Supplementary material for: Long-term outcomes of young, node-negative, chemotherapy-naïve, triple-negative breast cancer patients according to BRCA1 status
Source: BMC Med. 2024 Jan 9;22:9. doi: 10.1186/s12916-023-03233-7 (PMC10775514; doi:10.1186/s12916-023-03233-7)
Supplement: Supplementary file 5 — Additional file 5: Table S3. 3-, 5-, 10-, and 15-year overall survival rate, distant recurrence-free survival rate, and cumulative incidence of second primary tumors according to BRCA1 status. [file 12916_2023_3233_MOESM5_ESM.docx]

## **Table S3. 3-, 5-, 10-, and 15-year overall survival rate, distant recurrence-free survival rate, and cumulative incidence of second primary tumors according to *BRCA1* status**

|  | **No. of death** | **Overall survival**  **(95% CI)** | **No. of distant recurrence or death** | **Distant recurrence-free survival**  **(95% CI)** | **No. of second primary tumors** | **cumulative incidence of second primary tumors s**  **(95% CI)** |
| --- | --- | --- | --- | --- | --- | --- |
| ***BRCA1*-non-alteration (n = 124)** | | | | | | |
| 0 to 3 years | 20 | 83.9 (77.6-90.6) | 25 | 79.3 (72.4-86.9) | 5 | 4.0 (0.5-7.4) |
| 3 to 5 years | 8 | 77.4 (70.4-85.1) | 2 | 77.6 (70.5-85.4) | 2 | 5.6 (1.5-9.6) |
| 5 to 10 years | 4 | 74.2 (66.8-82.3) | 2 | 75.8 (68.5-83.9) | 4 | 8.9 (3.8-13.7) |
| 10 to 15 years | 5 | 70.1 (62.5-78.6) | 3 | 73.0 (65.4-81.5) | 6 | 13.8 (7.6-19.6) |
| **g*BRCA1*m (n = 105)** | | | | | | |
| 0 to 3 years | 12 | 88.6 (82.7-94.9) | 21 | 76.6 (68.3-86.0) | 19 | 18.1 (10.4-25.1) |
| 3 to 5 years | 18 | 71.4 (63.3-80.6) | 7 | 68.1 (58.9-78.7) | 8 | 25.7 (17.1-33.4) |
| 5 to 10 years | 5 | 66.7 (58.2-76.3) | 0 | 68.1 (58.9-78.7) | 16 | 41.0 (31.6-49.1) |
| 10 to 15 years | 5 | 61.8 (53.2-71.9) | 0 | 68.1 (58.9-78.7) | 5 | 45.8 (36.4-53.8) |
| **s*BRCA1*m (n = 21)** | | | | | | |
| 0 to 3 years | 4 | 81.0 (65.8-99.6) | 7 | 66.7 (49.3-90.2) | 0 | 0.0 (0.0-0.0) |
| 3 to 5 years | 2 | 71.4 (54.5-93.6) | 1 | 61.9 (44.3-86.6) | 0 | 0.0 (0.0-0.0) |
| 5 to 10 years | 1 | 66.7 (49.3-90.2) | 0 | 61.9 (44.3-86.6) | 1 | 4.8 (0.0-13.5) |
| 10 to 15 years | 0 | 66.7 (49.3-90.2) | 0 | 61.9 (44.3-86.6) | 0 | 4.8 (0.0-13.5) |
| **Tumor *BRCA1*-PM (n = 144)** | | | | | | |
| 0 to 3 years | 17 | 88.1 (83.0-93.6) | 22 | 84.5 (78.7-90.7) | 2 | 1.4 (0.0-3.3) |
| 3 to 5 years | 9 | 81.8 (75.7-88.4) | 4 | 81.6 (75.5-88.3) | 1 | 2.1 (0.0-4.4) |
| 5 to 10 years | 4 | 79.0 (72.6-86.0) | 3 | 79.4 (73.0-86.4) | 3 | 4.2 (0.9-7.5) |
| 10 to 15 years | 2 | 77.6 (71.0-84.7) | 2 | 77.9 (71.3-85.1) | 3 | 6.4 (2.3 -10.3) |

Abbreviations: CI, confidence interval; *BRCA1*-non-alteration, without germline *BRCA1* mutation, without somatic *BRCA1* mutation, and without tumor *BRCA1* promoter methylation; g*BRCA1*m, germline *BRCA1* mutation; s*BRCA1*m, somatic *BRCA1* mutation; tumor *BRCA1*-PM, tumor *BRCA1* promoter methylation.

Note that the total number of tumor *BRCA1* promoter methylated patients and *BRCA1*-non-altered patients were not 146 and 127, respectively, because five germline *BRCA2*-mutated patients were removed.
