# Supplementary material for: Long-term outcomes of young, node-negative, chemotherapy-naïve, triple-negative breast cancer patients according to BRCA1 status
Source: BMC Med. 2024 Jan 9;22:9. doi: 10.1186/s12916-023-03233-7 (PMC10775514; doi:10.1186/s12916-023-03233-7)
Supplement: Supplementary file 6 — Additional file 6: Table S4. 3-, 5-, 10-, and 15-year overall survival rate, distant recurrence-free survival rate, and cumulative incidence of second primary tumors according to BRCA1-like status. [file 12916_2023_3233_MOESM6_ESM.docx]

**Table S4. 3-, 5-, 10-, and 15-year overall survival rate, distant recurrence-free survival rate, and cumulative incidence of second primary tumors according to *BRCA1*-like status**

|  | **No. of death** | **Overall survival**  **(95% CI)** | **No. of distant recurrence or death** | **Distant recurrence-free survival**  **(95% CI)** | **No. of second primary tumors** | **cumulative incidence of second primary tumors s**  **(95% CI)** |
| --- | --- | --- | --- | --- | --- | --- |
| **Non-*BRCA1*-like (n = 48)** | | | | | | |
| 0 to 3 years | 5 | 89.6 (81.3-98.7) | 8 | 82.8 (72.7-94.4) | 2 | 4.2 ( 0.0- 9.7) |
| 3 to 5 years | 6 | 77.1 (66.1-89.9) | 4 | 73.9 (62.2-87.8) | 2 | 8.3 ( 0.2-15.8) |
| 5 to 10 years | 2 | 72.9 (61.4-86.6) | 0 | 73.9 (62.2-87.8) | 6 | 20.9 ( 8.8-31.3) |
| 10 to 15 years | 2 | 68.6 (56.7-83.1) | 0 | 73.9 (62.2-87.8) | 1 | 22.9 (10.5-33.7) |
| ***BRCA1*-like (n = 299)** | | | | | | |
| 0 to 3 years | 43 | 85.6 (81.7-89.7) | 59 | 79.5 (74.9-84.3) | 18 | 6.0 ( 3.3- 8.7) |
| 3 to 5 years | 24 | 77.6 (73.0-82.5) | 7 | 76.9 (72.2-82.0) | 6 | 8.0 ( 4.9-11.0) |
| 5 to 10 years | 8 | 74.9 (70.1-80.0) | 4 | 75.4 (70.5-80.6) | 18 | 14.1 (10.1-17.9) |
| 10 to 15 years | 9 | 71.8 (66.9-77.1) | 5 | 73.3 (68.2-78.7) | 10 | 17.5 (13.1-21.6) |

Abbreviations: CI, confidence interval.
