# Supplementary material for: Long-term outcomes of young, node-negative, chemotherapy-naïve, triple-negative breast cancer patients according to BRCA1 status
Source: BMC Med. 2024 Jan 9;22:9. doi: 10.1186/s12916-023-03233-7 (PMC10775514; doi:10.1186/s12916-023-03233-7)
Supplement: Supplementary file 7 — Additional file 7: Table S5. Hazard ratios for overall survival according to BRCA1 status, based on multiple-imputed data. [file 12916_2023_3233_MOESM7_ESM.docx]

## **Table S5. Hazard ratios for overall survival according to *BRCA1* status, based on multiple-imputed data**

|  | **All patients**  **(n = 479) ^e^** | **All patients (additional adjustment for second primary tumors)** | **Patients diagnosed from 1989-1997**  **(n = 422) ^e^** | **Patients with ER and PR < 1%**  **(n = 455) ^e^** | **Patients with *BRCA1*-like tumors**  **(n = 401)^e^** |
| --- | --- | --- | --- | --- | --- |
|  | **HR (95% CI)** | **HR (95% CI)** | **HR (95% CI)** | **HR (95% CI)** | **HR (95% CI)** |
| **Univariable** | | | | | |
| *BRCA1-*non-alteration | 1.00 (referent) | NA | 1.00 (referent) | 1.00 (referent) | 1.00 (referent) |
| g*BRCA1*m 0-3 years ^a^ | 0.73 (0.36-1.47) | NA | 0.76 (0.36-1.60) | 0.66 (0.31-1.41) | 0.61 (0.27-1.37) |
| g*BRCA1*m 4-15 years ^a^ | 2.00 (1.15-3.47) | NA | 1.75 (0.97-3.15) | 2.09 (1.17-3.73) | 2.05 (1.08-3.87) |
| s*BRCA1*m | 1.17 (0.51-2.67) | NA | 1.24 (0.54-2.87) | 1.19 (0.52-2.71) | 0.85 (0.29-2.49) |
| Tumor *BRCA1*-PM | 0.72 (0.45-1.15) | NA | 0.64 (0.39-1.07) | 0.75 (0.46-1.22) | 0.73 (0.44-1.20) |
| **Multivariable** | | | | | |
| ***BRCA1* status** | | | | | |
| *BRCA1-*non-alteration | 1.00 (referent) | 1.00 (referent) | 1.00 (referent) | 1.00 (referent) | 1.00 (referent) |
| g*BRCA1*m 0-3 years ^a^ | 0.75 (0.36-1.53) | 0.60 (0.29-1.27) | 0.78 (0.36-1.69) | 0.68 (0.31-1.47) | 0.64 (0.28-1.47) |
| g*BRCA1*m 4-15 years ^a^ | 2.11 (1.18-3.75) | 1.43 (0.77-2.66) | 1.82 (0.98-3.37) | 2.24 (1.23-4.09) | 2.12 (1.10-4.11) |
| s*BRCA1*m | 0.96 (0.42-2.21) | 1.02 (0.44-2.38) | 1.05 (0.45-2.45) | 1.02 (0.45-2.33) | 0.79 (0.27-2.34) |
| Tumor *BRCA1*-PM | 1.19 (0.65-2.16) | 1.25 (0.68-2.28) | 1.16 (0.62-2.18) | 1.16 (0.62-2.18) | 1.31 (0.68-2.51) |
| **sTILs (per 10% increment)** | 0.84 (0.78-0.90) | 0.82 (0.76-0.89) | 0.84 (0.78-0.91) | 0.84 (0.78-0.91) | 0.85 (0.77-0.93) |
| **Interaction term** | | | | | |
| sTILs by tumor *BRCA1*-PM status | 0.82 (0.68-0.98) | 0.83 (0.69-1.00) | 0.77 (0.62-0.97) | 0.84 (0.70-1.02) | 0.77 (0.60-0.99) |
| **Tumor size** | | | | | |
| ≤ 20 mm | 1.00 (referent) | 1.00 (referent) | 1.00 (referent) | 1.00 (referent) | 1.00 (referent) |
| > 20mm | 1.55 (1.07-2.23) | 1.59 (1.10-2.29) | 1.57 (1.07-2.33) | 1.48 (1.01-2.18) | 1.53 (1.00-2.32) |
| **Tumor grade** | | | | | |
| Grade 1 or grade 2 | 1.00 (referent) | 1.00 (referent) | 1.00 (referent) | 1.00 (referent) | 1.00 (referent) |
| Grade 3 | 1.45 (0.86-2.45) | 1.40 (0.83-2.37) | 1.51 (0.86-2.64) | 1.41 (0.82-2.43) | 1.65 (0.88-3.10) |

(Continued)

|  | **All patients**  **(n = 479) ^e^** | **All patients (additional adjustment for second primary tumors)** | **Patients diagnosed from 1989-1997**  **(n = 422) ^e^** | **Patients with ER and PR < 1%**  **(n = 455) ^e^** | **Patients with *BRCA1*-like tumors (n = 401)^e^** |
| --- | --- | --- | --- | --- | --- |
|  | **HR (95% CI)** | **HR (95% CI)** | **HR (95% CI)** | **HR (95% CI)** | **HR (95% CI)** |
| **Histological subtypes** | | | | | |
| Carcinoma of no special type | 1.00 (referent) | 1.00 (referent) | 1.00 (referent) | 1.00 (referent) | 1.00 (referent) |
| Metaplastic carcinoma | 0.36 (0.13-0.99) | 0.37 (0.13-1.04) | 0.39 (0.14-1.10) | 0.44 (0.16-1.23) | 0.33 (0.10-1.10) |
| Other histological types ^b^ | 0.62 (0.19-2.09) | 0.66 (0.20-2.22) | 0.61 (0.14-2.59) | 0.65 (0.19-2.20) | 0.66 (0.15-2.89) |
| **Lymphovascular invasion** | | | | | |
| No | 1.00 (referent) | 1.00 (referent) | 1.00 (referent) | 1.00 (referent) | 1.00 (referent) |
| Yes | 2.40 (1.55-3.72) | 2.72 (1.75-4.24) | 2.56 (1.61-4.08) | 2.42 (1.54-3.78) | 2.45 (1.46-4.11) |
| **Locoregional treatment** | | | | | |
| Lumpectomy and radiotherapy | 1.00 (referent) | 1.00 (referent) | 1.00 (referent) | 1.00 (referent) | 1.00 (referent) |
| Mastectomy alone | 1.35 (0.92-2.00) | 1.43 (0.97-2.11) | 1.36 (0.89-2.07) | 1.29 (0.85-1.94) | 1.32 (0.83-2.11) |
| Other treatment ^c^ | 1.61 (0.87-3.01) | 1.78 (0.95-3.33) | 1.37 (0.69-2.69) | 1.71 (0.91-3.20) | 1.65 (0.81-3.40) |
| **Second primary tumors ^d^** | | | | | |
| No | NA | 1.00 (referent) | NA | NA | NA |
| Yes | NA | 4.28 (2.57-7.15) | NA | NA | NA |

Abbreviations: HR, hazard ratio; CI, confidence interval; *BRCA1*-non-alteration, without germline *BRCA1* mutation, without somatic *BRCA1* mutation and without tumor *BRCA1* promoter methylation; g*BRCA1*m, germline *BRCA1* mutation; s*BRCA1*m, somatic *BRCA1* mutation; tumor *BRCA1*-PM, tumor *BRCA1* promoter methylation; sTILs, stromal tumor infiltrating lymphocytes; ER, estrogen receptor; PR, progesterone receptor; NA, not applicable.

^a^ Hazard ratios for g*BRCA1*m was estimated for the first three years and from the fourth year onwards separately for overall survival because of non-proportional hazards.

^b^ Other histological subtypes include adenoid cystic carcinoma, apocrine carcinoma, ductal-lobular carcinoma, invasive cribriform carcinoma, invasive papillary carcinoma, invasive lobular carcinoma, invasive micropapillary carcinoma.

^c^ Other treatment include lumpectomy alone, mastectomy and radiotherapy, and unspecified surgery with and without radiotherapy.

^d^ Second primary tumors (yes/ no) was a time-varying covariate, i.e. with the value of 0 until the time when a second primary tumor occurred and with the value of 1 after that time. We only included second primary tumors as an additional adjustment into the multivariable Cox regression model for overall survival, therefore the rest models did not have a hazard ratio for second primary tumors.

^e^ The number of patients was the median number across the imputed datasets because the numbers of different imputed datasets could be different as germline *BRCA2-*mutated patients were removed from analysis (imputed variable), and/or patients with *BRCA1*-like tumors (imputed variable) were selected for sensitivity analysis.
