# Supplementary material for: Long-term outcomes of young, node-negative, chemotherapy-naïve, triple-negative breast cancer patients according to BRCA1 status
Source: BMC Med. 2024 Jan 9;22:9. doi: 10.1186/s12916-023-03233-7 (PMC10775514; doi:10.1186/s12916-023-03233-7)
Supplement: Supplementary file 8 — Additional file 8: Table S6. Hazard ratios for distant recurrence-free survival according to BRCA1 status, based on multiple-imputed data. [file 12916_2023_3233_MOESM8_ESM.docx]

## **Table S6. Hazard ratios for distant recurrence-free survival according to *BRCA1* status, based on multiple-imputed data**

|  | **All patients**  **(n = 479) ^c^** | **Patients diagnosed from 1989-1997**  **(n = 422) ^c^** | **Patients with ER and PR < 1%**  **(n = 455) ^c^** | **Patients with *BRCA1*-like tumors**  **(n = 402)^c^** |
| --- | --- | --- | --- | --- |
|  | **HR (95% CI)** | **HR (95% CI)** | **HR (95% CI)** | **HR (95% CI)** |
| **Univariable** | | | | |
| *BRCA1*-non-alteration | 1.00 (referent) | 1.00 (referent) | 1.00 (referent) | 1.00 (referent) |
| g*BRCA1*m | 1.29 (0.79-2.11) | 1.26 (0.74-2.13) | 1.30 (0.78-2.19) | 1.28 (0.74-2.21) |
| s*BRCA1*m | 1.52 (0.67-3.44) | 1.66 (0.72-3.80) | 1.58 (0.70-3.58) | 1.30 (0.49-3.46) |
| Tumor *BRCA1*-PM | 0.77 (0.47-1.27) | 0.72 (0.42-1.22) | 0.81 (0.49-1.36) | 0.78 (0.46-1.34) |
| **Multivariable** | | | | |
| ***BRCA1* status** | | | | |
| *BRCA1*-non-alteration | 1.00 (referent) | 1.00 (referent) | 1.00 (referent) | NA ^d^ |
| g*BRCA1*m | 1.34 (0.78-2.28) | 1.28 (0.72-2.27) | 1.35 (0.77-2.35) |  |
| s*BRCA1*m | 1.30 (0.55-3.06) | 1.53 (0.64-3.66) | 1.39 (0.59-3.29) |  |
| Tumor *BRCA1*-PM | 0.88 (0.51-1.51) | 0.83 (0.46-1.47) | 0.92 (0.52-1.63) |  |
| **sTILs (per 10% increment)** | 0.74 (0.68-0.80) | 0.73 (0.66-0.80) | 0.75 (0.68-0.81) | NA ^d^ |
| **Tumor size** | | | | |
| ≤ 20 mm | 1.00 (referent) | 1.00 (referent) | 1.00 (referent) | NA ^d^ |
| > 20mm | 1.49 (1.00-2.20) | 1.52 (1.00-2.33) | 1.52 (1.01-2.30) |  |
| **Tumor grade** | | | | |
| Grade 1 or grade 2 |  |  |  | NA ^d^ |
| Grade 3 | 1.21 (0.72-2.03) | 1.33 (0.76-2.33) | 1.19 (0.70-2.05) |  |
| **Histological subtypes** | | | | |
| Carcinoma of no special type | 1.00 (referent) | 1.00 (referent) | 1.00 (referent) | NA ^d^ |
| Metaplastic carcinoma | 0.20 (0.05-0.83) | 0.21 (0.05-0.90) | 0.25 (0.06-1.04) |  |
| Other histological types ^a^ | 0.37 (0.09-1.60) | 0.27 (0.04-2.05) | 0.38 (0.09-1.65) |  |
| **Lymphovascular invasion** | | | | |
| No | 1.00 (referent) | 1.00 (referent) | 1.00 (referent) | NA ^d^ |
| Yes | 2.41 (1.54-3.79) | 2.51 (1.55-4.06) | 2.47 (1.55-3.94) |  |
| **Locoregional treatment** | | | | |
| Lumpectomy and radiotherapy | 1.00 (referent) | 1.00 (referent) | 1.00 (referent) | NA ^d^ |
| Mastectomy alone | 1.54 (1.01-2.34) | 1.57 (1.00-2.47) | 1.49 (0.96-2.33) |  |
| Other treatment ^b^ | 1.82 (0.96-3.47) | 1.54 (0.76-3.11) | 1.89 (0.99-3.61) |  |

Abbreviations: HR, hazard ratio; CI, confidence interval; *BRCA1*-non-alteration, without germline *BRCA1* mutation, without somatic *BRCA1* mutation, and without tumor *BRCA1* promoter methylation; g*BRCA1*m, germline *BRCA1* mutation; s*BRCA1*m, somatic *BRCA1* mutation; tumor *BRCA1*-PM, tumor *BRCA1* promoter methylation; sTILs, stromal tumor infiltrating lymphocytes; ER, estrogen receptor; PR, progesterone receptor; NA, not applicable.

^a^ Other histological subtypes include adenoid cystic carcinoma, apocrine carcinoma, ductal-lobular carcinoma, invasive cribriform carcinoma, invasive papillary carcinoma, invasive lobular carcinoma, invasive micropapillary carcinoma.

^b^ Other treatment include lumpectomy alone, mastectomy and radiotherapy, and unspecified surgery with and without radiotherapy.

^c^ The number of patients was the median number across the imputed datasets because the numbers of different imputed datasets could be different as germline *BRCA2-*mutated patients were removed from analysis (imputed variable), and/or patients with *BRCA1*-like tumors (imputed variable) were selected for sensitivity analysis.

^d^ The multivariable model did not converged, therefore no results were computed.
