# Supplementary material for: Long-term outcomes of young, node-negative, chemotherapy-naïve, triple-negative breast cancer patients according to BRCA1 status
Source: BMC Med. 2024 Jan 9;22:9. doi: 10.1186/s12916-023-03233-7 (PMC10775514; doi:10.1186/s12916-023-03233-7)
Supplement: Supplementary file 9 — Additional file 9: Table S7. Subdistribution hazard ratios for second primary tumors according to BRCA1 status, based on multiple-imputed data, using Fine and Gray competing risk models with distant recurrence and death as competing events. [file 12916_2023_3233_MOESM9_ESM.docx]

## **Table S7. Subdistribution hazard ratios for second primary tumors according to *BRCA1* status, based on multiple-imputed data, using Fine and Gray competing risk models with distant recurrence and death as competing events**

|  | **All patients**  **(n = 478)^c^** | **Patients diagnosed from 1989-1997**  **(n = 421) ^c^** | **Patients with ER and PR < 1%**  **(n = 454) ^c^** | **Patients with *BRCA1*-like tumors**  **(n = 402) ^c^** |
| --- | --- | --- | --- | --- |
|  | **sHR (95% CI)** | **sHR (95% CI)** | **sHR (95% CI)** | **sHR (95% CI)** |
| **Univariable** | | | | |
| *BRCA1*-non-alteration | 1.00 (referent) | 1.00 (referent) | 1.00 (referent) | 1.00 (referent) |
| g*BRCA1*m | 4.00 (2.34-6.86) | 3.58 (2.06-6.20) | 3.64 (2.09-6.32) | 3.20 (1.75-5.85) |
| s*BRCA1*m | 0.49 (0.07-3.41) | 0.48 (0.07-3.32) | 0.46 (0.07-3.22) | 0.45 (0.06-3.29) |
| Tumor *BRCA1*-PM | 0.46 (0.21-1.02) | 0.42 (0.18-0.95) | 0.43 (0.19-0.99) | 0.39 (0.17-0.91) |
| **Multivariable** | | | | |
| ***BRCA1* status** | | | | |
| *BRCA1*-non-alteration | 1.00 (referent) | 1.00 (referent) | 1.00 (referent) | 1.00 (referent) |
| g*BRCA1*m | 4.04 (2.29-7.13) | 3.74 (2.05-6.81) | 3.62 (2.02-6.48) | 3.21 (1.69-6.12) |
| s*BRCA1*m | 0.49 (0.07-3.47) | 0.47 (0.07-3.35) | 0.46 (0.06-3.30) | 0.43 (0.06-3.24) |
| Tumor *BRCA1*-PM | 0.42 (0.19-0.95) | 0.37 (0.16-0.87) | 0.40 (0.17-0.93) | 0.36 (0.15-0.85) |
| **sTILs (per 10% increment)** | 1.09 (1.02-1.16) | 1.10 (1.02-1.17) | 1.10 (1.03-1.17) | 1.07 (0.99-1.16) |
| **Tumor size** | | | | |
| ≤20 mm | 1.00 (referent) | 1.00 (referent) | 1.00 (referent) | 1.00 (referent) |
| > 20mm | 1.01 (0.65-1.58) | 1.01 (0.63-1.63) | 0.96 (0.60-1.52) | 0.99 (0.59-1.68) |
| **Tumor grade** | | | | |
| Grade 1 or grade 2 | 1.00 (referent) | 1.00 (referent) | 1.00 (referent) | 1.00 (referent) |
| Grade 3 | 1.52 (0.71-3.30) | 1.44 (0.66-3.12) | 1.40 (0.64-3.08) | 1.75 (0.67-4.58) |
| **Histological subtypes** | | | | |
| Carcinoma of no special type | 1.00 (referent) | 1.00 (referent) | 1.00 (referent) | 1.00 (referent) |
| Metaplastic carcinoma | 0.97 (0.37-2.58) | 0.91 (0.26-3.26) | 1.15 (0.42-3.18) | 1.20 (0.32-4.42) |
| Other histological types ^a^ | 1.54 (0.40-5.93) | 1.57 (0.41-6.07) | 1.47 (0.38-5.72) | 1.29 (0.23-7.31) |
| **Lymphovascular invasion** | | | | |
| No | 1.00 (referent) | 1.00 (referent) | 1.00 (referent) | 1.00 (referent) |
| Yes | 0.45 (0.18-1.13) | 0.47 (0.19-1.18) | 0.45 (0.18-1.14) | 0.51 (0.16-1.59) |
| **Locoregional treatment** | | | | |
| Lumpectomy and radiotherapy | 1.00 (referent) | 1.00 (referent) | 1.00 (referent) | 1.00 (referent) |
| Mastectomy alone | 0.89 (0.56-1.41) | 0.83 (0.51-1.35) | 1.05 (0.64-1.71) | 0.93 (0.52-1.64) |
| Other treatment ^b^ | 0.57 (0.22-1.46) | 0.45 (0.14-1.43) | 0.66 (0.25-1.69) | 0.51 (0.16-1.65) |

Abbreviations: sHR, subdistribution hazard ratio; CI, confidence interval; *BRCA1*-non-alteration, without germline *BRCA1* mutation, without somatic *BRCA1* mutation, and without tumor *BRCA1* promoter methylation; g*BRCA1*m, germline *BRCA1* mutation; s*BRCA1*m, somatic *BRCA1* mutation; tumor *BRCA1*-PM, tumor *BRCA1* promoter methylation; sTILs, stromal tumor infiltrating lymphocytes; ER, estrogen receptor; PR, progesterone receptor.

^a^ Other histological subtypes include adenoid cystic carcinoma, apocrine carcinoma, ductal-lobular carcinoma, invasive cribriform carcinoma, invasive papillary carcinoma, invasive lobular carcinoma, invasive micropapillary carcinoma.

^b^ Other treatment include lumpectomy alone, mastectomy and radiotherapy, and unspecified surgery with and without radiotherapy.

^c^ The number of patients was the median number across the imputed datasets because the numbers of different imputed datasets could be different as germline *BRCA2-*mutated patients were removed from analysis (imputed variable), and/or patients with *BRCA1*-like tumors (imputed variable) were selected for sensitivity analysis.
