# Supplementary material for: Long-term outcomes of young, node-negative, chemotherapy-naïve, triple-negative breast cancer patients according to BRCA1 status
Source: BMC Med. 2024 Jan 9;22:9. doi: 10.1186/s12916-023-03233-7 (PMC10775514; doi:10.1186/s12916-023-03233-7)
Supplement: Supplementary file 10 — Additional file 10: Table S8. Hazard ratios for second primary tumors according to BRCA1 status, based on multiple-imputed data, using cause-specific competing risk models with distant recurrence and death as competing events. [file 12916_2023_3233_MOESM10_ESM.docx]

## **Table S8. Hazard ratios for second primary tumors according to *BRCA1* status, based on multiple-imputed data, using cause-specific competing risk models with distant recurrence and death as competing events**

|  | **All patients**  **(N = 478)^c^** | **Patients diagnosed from 1989-1997**  **(N = 421) ^c^** | **Patients with ER and PR < 1%**  **(N = 454) ^c^** | **Patients with *BRCA1*-like tumors**  **(N = 401) ^c^** |
| --- | --- | --- | --- | --- |
|  | **HR (95% CI)** | **HR (95% CI)** | **HR (95% CI)** | **HR (95% CI)** |
| **Univariable** | | | | |
| *BRCA1*-non-alteration | 1.00 (referent) | 1.00 (referent) | 1.00 (referent) | 1.00 (referent) |
| g*BRCA1*m | 5.39 (3.08-9.43) | 4.84 (2.75-8.52) | 4.88 (2.76-8.64) | 4.30 (2.29-8.09) |
| s*BRCA1*m | 0.48 (0.07-3.57) | 0.49 (0.07-3.59) | 0.45 (0.06-3.36) | 0.41 (0.05-3.27) |
| Tumor *BRCA1*-PM | 0.42 (0.18-0.95) | 0.37 (0.16-0.87) | 0.39 (0.17-0.92) | 0.36 (0.15-0.87) |
| **Multivariable** | | | | |
| ***BRCA1* status** | | | | |
| *BRCA1*-non-alteration | 1.00 (referent) | 1.00 (referent) | 1.00 (referent) | 1.00 (referent) |
| g*BRCA1*m | 5.33 (3.02-9.40) | 4.91 (2.75-8.77) | 4.73 (2.65-8.47) | 4.32 (2.25-8.29) |
| s*BRCA1*m | 0.44 (0.06-3.37) | 0.44 (0.06-3.35) | 0.42 (0.06-3.24) | 0.38 (0.05-3.02) |
| Tumor *BRCA1*-PM | 0.41 (0.18-0.93) | 0.35 (0.15-0.84) | 0.38 (0.16-0.89) | 0.35 (0.14-0.85) |
| **sTILs (per 10% increment)** | 1.03 (0.96-1.10) | 1.04 (0.96-1.12) | 1.04 (0.97-1.12) | 1.02 (0.94-1.10) |
| **Tumor size** | | | | |
| ≤ 20 mm | 1.00 (referent) | 1.00 (referent) | 1.00 (referent) | 1.00 (referent) |
| > 20mm | 1.10 (0.67-1.81) | 1.11 (0.65-1.91) | 1.04 (0.62-1.76) | 1.10 (0.62-1.96) |
| **Tumor grade** |  |  |  |  |
| Grade 1 or grade 2 | 1.00 (referent) | 1.00 (referent) | 1.00 (referent) | 1.00 (referent) |
| Grade 3 | 1.57 (0.71-3.47) | 1.53 (0.69-3.39) | 1.40 (0.63-3.14) | 1.94 (0.69-5.47) |
| **Histological subtypes** | | | | |
| Carcinoma of no special type | 1.00 (referent) | 1.00 (referent) | 1.00 (referent) | 1.00 (referent) |
| Metaplastic carcinoma | 0.72 (0.25-2.06) | 0.68 (0.20-2.28) | 0.91 (0.31-2.64) | 0.85 (0.25-2.96) |
| Other histological types ^a^ | 1.24 (0.36-4.30) | 1.24 (0.35-4.35) | 1.19 (0.34-4.18) | 0.91 (0.15-5.40) |
| **Lymphovascular invasion** | | | | |
| No | 1.00 (referent) | 1.00 (referent) | 1.00 (referent) | 1.00 (referent) |
| Yes | 0.63 (0.24-1.64) | 0.65 (0.25-1.71) | 0.63 (0.24-1.65) | 0.70 (0.23-2.20) |
| **Locoregional treatment** | | | | |
| Lumpectomy and radiotherapy | 1.00 (referent) | 1.00 (referent) | 1.00 (referent) | 1.00 (referent) |
| Mastectomy alone | 0.98 (0.58-1.67) | 0.94 (0.53-1.64) | 1.11 (0.64-1.93) | 1.01 (0.55-1.88) |
| Other treatment ^b^ | 0.69 (0.24-1.98) | 0.54 (0.16-1.86) | 0.83 (0.28-2.41) | 0.65 (0.19-2.29) |

Abbreviations: HR, hazard ratio; CI, confidence interval; *BRCA1*-non-alteration, tumor without germline *BRCA1* mutation, somatic *BRCA1* mutation or tumor *BRCA1* promoter methylation; g*BRCA1*m, germline *BRCA1* mutation; s*BRCA1*m, somatic *BRCA1* mutation; tumor *BRCA1*-PM, tumor *BRCA1* promoter methylation; sTILs, stromal tumor infiltrating lymphocytes; ER, estrogen receptor; PR, progesterone receptor.

^a^ Other histological subtypes include adenoid cystic carcinoma, apocrine carcinoma, ductal-lobular carcinoma, invasive cribriform carcinoma, invasive papillary carcinoma, invasive lobular carcinoma, invasive micropapillary carcinoma.

^b^ Other treatment include lumpectomy alone, mastectomy and radiotherapy, and unspecified surgery with and without radiotherapy.

^c^ The number of patients was the median number across the imputed datasets because the numbers of different imputed datasets could be different as germline *BRCA2-*mutated patients were removed from analysis (imputed variable), and/or patients with *BRCA1*-like tumors (imputed variable) were selected for sensitivity analysis.
