# Supplementary material for: Long-term outcomes of young, node-negative, chemotherapy-naïve, triple-negative breast cancer patients according to BRCA1 status
Source: BMC Med. 2024 Jan 9;22:9. doi: 10.1186/s12916-023-03233-7 (PMC10775514; doi:10.1186/s12916-023-03233-7)
Supplement: Supplementary file 11 — Additional file 11. Univariable (subdistribution) hazard ratios according to BRCA1-like status. [file 12916_2023_3233_MOESM11_ESM.docx]

## **Table S9. Univariable (subdistribution) hazard ratios according to *BRCA1*-like status**

|  | **Overall survival**  **HR (95% CI)** | **Distant recurrence-free survival**  **HR (95% CI)** | **Second primary tumors (Fine and Gray model) ^a^**  **sHR (95% CI)** | **Second primary tumors (cause-specific model) ^b^**  **HR (95% CI)** |
| --- | --- | --- | --- | --- |
| **Based on multiple-imputed data** | | | | |
| *BRCA1*-like status | | | | |
| Non-*BRCA1*-like | 1.00 (referent) | 1.00 (referent) | 1.00 (referent) | 1.00 (referent) |
| *BRCA1*-like | 0.88 (0.52-1.47) | 1.02 (0.56-1.86) | 0.78 (0.41-1.48) | 0.72 (0.38-1.39) |
| **Based on cases with complete information** | | | | |
| *BRCA1*-like status | | | | |
| Non-*BRCA1*-like | 1.00 (referent) | 1.00 (referent) | 1.00 (referent) | 1.00 (referent) |
| *BRCA1*-like | 0.92 (0.53-1.59) | 1.03 (0.56-1.89) | 0.74 (0.40-1.39) | 0.73 (0.38-1.40) |

Abbreviations: HR, hazard ratio; sHR, subdistribution hazard ratio; CI, confidence interval.

^a^ Fine and Gray competing risk models were used to calculate subdistribution hazard ratios. Second primary tumors were events of interest; death and distant recurrence were competing events.

^b^ Cause-specific competing risk models were used to calculate hazard ratios. Second primary tumors were events of interest; death and distant recurrence were competing events (censored).
