# Supplementary material for: Long-term outcomes of young, node-negative, chemotherapy-naïve, triple-negative breast cancer patients according to BRCA1 status
Source: BMC Med. 2024 Jan 9;22:9. doi: 10.1186/s12916-023-03233-7 (PMC10775514; doi:10.1186/s12916-023-03233-7)
Supplement: Supplementary file 12 — Additional file 12. (subdistribution) Hazard ratios according to BRCA1 status, based on cases with complete information. [file 12916_2023_3233_MOESM12_ESM.docx]

## **Table S10. (subdistribution) Hazard ratios according to *BRCA1* status, based on cases with complete information**

|  | **Overall survival**  **HR (95% CI)** | **Overall survival, with additional adjustment for second primary tumors**  **HR (95% CI)** | **Distant recurrence-free survival**  **HR (95% CI)** | **Second primary tumors (Fine and Gray model) ^f^**  **sHR (95% CI)** | **Second primary tumors (cause-specific model) ^g^**  **HR (95% CI)** |
| --- | --- | --- | --- | --- | --- |
| **Univariable** | | | | | |
| *BRCA1-*non-alteration | 1.00 (referent) | NA | 1.00 (referent) | 1.00 (referent) | 1.00 (referent) |
| g*BRCA1*m 0-3 years ^a^ | 0.68 (0.35-1.36) | NA | 1.22 (0.74-2.03) | 4.28 (2.52-7.28) | 5.01 (2.87-8.75) |
| g*BRCA1*m 4-15 years ^a^ | 2.04 (1.18-3.53) | NA |  |  |  |
| s*BRCA1*m | 1.15 (0.51-2.57) | NA | 1.59 (0.73-3.45) | 0.33 (0.05-2.38) | 0.39 (0.05-2.91) |
| Tumor *BRCA1*-PM | 0.73 (0.45-1.17) | NA | 0.78 (0.48-1.28) | 0.44 (0.20-0.98) | 0.42 (0.19-0.95) |
| **Multivariable** | | | | | |
| ***BRCA1* status** | | | | | |
| *BRCA1-*non-alteration | 1.00 (referent) | 1.00 (referent) | 1.00 (referent) | 1.00 (referent) | 1.00 (referent) |
| g*BRCA1*m 0-3 years ^a^ | 0.73 (0.37-1.47) | 0.60 (0.30-1.21) | 1.28 (0.75-2.18) | 4.25 (2.44-7.39) | 4.86 (2.77-8.54) |
| g*BRCA1*m 4-15 years ^a^ | 2.20 (1.25-3.86) | 1.52 (0.83-2.82) |  |  |  |
| s*BRCA1*m | 0.97 (0.43-2.21) | 1.06 (0.49-2.28) | 1.40 (0.63-3.10) | 0.31 (0.04-2.18) | 0.33 (0.04-2.49) |
| Tumor *BRCA1*-PM | 1.50 (0.84-2.70) | 1.59 (0.88-2.84) | 0.98 (0.59-1.63) | 0.39 (0.17-0.89) | 0.40 (0.18-0.89) |
| **sTILs (per 10% increment)** | 0.84 (0.77-0.90) | 0.82 (0.76-0.88) | 0.71 (0.65-0.78) | 1.10 (1.03-1.17) | 1.03 (0.96-1.11) |
| **Interaction term ^b^** | | | | | |
| sTILs (per 10% increment) by tumor *BRCA1*-PM | 0.77 (0.63-0.94) | 0.79 (0.64-0.97) | NA | NA | NA |
| **Tumor size** | | | | | |
| ≤ 20 mm | 1.00 (referent) | 1.00 (referent) | 1.00 (referent) | 1.00 (referent) | 1.00 (referent) |
| > 20mm | 1.76 (1.18-2.60) | 1.75 (1.16-2.63) | 1.63 (1.07-2.49) | 1.18 (0.76-1.85) | 1.26 (0.76-2.08) |
| **Tumor grade** | | | | | |
| Grade 1 or grade 2 | 1.00 (referent) | 1.00 (referent) | 1.00 (referent) | 1.00 (referent) | 1.00 (referent) |
| Grade 3 | 1.12 (0.63-1.99) | 1.05 (0.60-1.84) | 0.94 (0.53-1.67) | 1.99 (0.71-5.54) | 2.00 (0.71-5.61) |
| **Histological subtypes** | | | | | |
| Carcinoma of no special type | 1.00 (referent) | 1.00 (referent) | 1.00 (referent) | 1.00 (referent) | 1.00 (referent) |
| Metaplastic carcinoma | 0.26 (0.08-0.84) | 0.27 (0.07-0.99) | 0.20 (0.05-0.83) | 0.74 (0.25-2.13) | 0.53 (0.16-1.73) |
| Other histological types ^c^ | 0.63 (0.19-2.14) | 0.66 (0.24-1.82) | 0.37 (0.09-1.59) | 1.11 (0.20-6.19) | 0.90 (0.21-3.87) |

(Continued)

|  | **Overall survival**  **HR (95% CI)** | **Overall survival, with additional adjustment for second primary tumors**  **HR (95% CI)** | **Distant recurrence-free survival**  **HR (95% CI)** | **Second primary tumors (Fine and Gray model) ^f^**  **sHR (95% CI)** | **Second primary tumors (cause-specific model) ^g^**  **HR (95% CI)** |
| --- | --- | --- | --- | --- | --- |
| **Lymphovascular invasion** | | | | | |
| No | 1.00 (referent) | 1.00 (referent) | 1.00 (referent) | 1.00 (referent) | 1.00 (referent) |
| Yes | 2.40 (1.49-3.87) | 2.69 (1.62-4.47) | 2.44 (1.49-3.99) | 0.43 (0.16-1.20) | 0.57 (0.20-1.57) |
| **Locoregional treatment** |  |  |  |  |  |
| Lumpectomy and radiotherapy | 1.00 (referent) | 1.00 (referent) | 1.00 (referent) | 1.00 (referent) | 1.00 (referent) |
| Mastectomy alone | 1.34 (0.88-2.03) | 1.41 (0.92-2.14) | 1.56 (0.99-2.45) | 0.94 (0.59-1.49) | 1.03 (0.60-1.75) |
| Other treatment ^d^ | 1.58 (0.79-3.17) | 1.81 (0.81-4.05) | 1.77 (0.86-3.65) | 0.61 (0.24-1.51) | 0.72 (0.25-2.02) |
| **Second primary tumors ^e^** | | | | | |
| No | NA | 1.00 (referent) | NA | NA | NA |
| Yes | NA | 3.71 (2.08-6.61) | NA | NA | NA |

Abbreviations: HR, hazard ratio; sHR, subdistribution hazard ratio; CI, confidence interval; *BRCA1*-non-alteration, without germline *BRCA1* mutation, without somatic *BRCA1* mutation and without tumor *BRCA1* promoter methylation; g*BRCA1*m, germline *BRCA1* mutation; s*BRCA1*m, somatic *BRCA1* mutation; tumor *BRCA1*-PM, tumor *BRCA1* promoter methylation; sTILs, stromal tumor infiltrating lymphocytes; ER, estrogen receptor; PR, progesterone receptor; NA, not applicable.

^a^ Hazard ratios for g*BRCA1*m was estimated for the first three years and from the fourth year onwards separately for overall survival because of non-proportional hazards.

^b^ Interaction terms between germline *BRCA1* mutation or somatic *BRCA1* mutation, and stromal tumor infiltrating lymphocytes were not significant, thus were not included in the final model for overall survival. None interaction terms were significant in the models for distant recurrence-free survival or cumulative incidence of second primary tumor.

^c^ Other histological subtypes include adenoid cystic carcinoma, apocrine carcinoma, ductal-lobular carcinoma, invasive cribriform carcinoma, invasive papillary carcinoma, invasive lobular carcinoma, invasive micropapillary carcinoma.

^d^ Other treatment include lumpectomy alone, mastectomy and radiotherapy, and unspecified surgery with and without radiotherapy.

^e^ Second primary tumors (yes/ no) was a time-varying covariate, i.e. with the value of 0 until the time when a second primary tumor occurred and with the value of 1 after that time.

^f^ Fine and Gray competing risk models were used to calculate subdistribution hazard ratios. Second primary tumors were events of interest; death and distant recurrence were competing events.

^g^ Cause-specific competing risk models were used to calculate hazard ratios. Second primary tumors were events of interest; death and distant recurrence were competing events (censored).
